# Application of the antitussive agents oxelaidin and butamirate as anti-glioma agents

**DOI:** 10.1038/s41598-021-89238-9

**Published:** 2021-05-12

**Authors:** Sook-Ja Lee, Seon-Yong Yeom, Jee-Young Lee, Chaehwa Park

**Affiliations:** 1 Research Institute for Future Medicine, Samsung Medical Center, Sungkyunkwan University School of Medicine, Irwon-dong, Seoul, 06351 Korea; 2 Department of Medicine, Samsung Medical Center, Sungkyunkwan University School of Medicine, Seoul, Korea; 3 Daegu-Gyeongbuk Medical Innovation Foundation, New Drug Development Center, Daegu, Korea

**Keywords:** Cancer, Molecular biology

## Abstract

Glioblastoma (GBM) is an aggressive brain tumor with a strong tendency of relapse and resistance to chemotherapy, but we currently lack non-toxic agents that effectively treat GBM. In this study, high-throughput screening of FDA-approved drugs was performed to identify safe and effective molecules and test their effect on GBM cell lines, LN229, U87 and T98G. Cough suppressants, oxelaidin and butamirate inhibited GBM growth. A Ras family GTPase, Ras-related associated with diabetes (RRAD), contributes to activation of STAT3, which is essential for survival and growth of many cancer types. Interestingly, oxelaidin and butamirate did not affect proliferation in RRAD negative GBM cells. Docking simulation analyses revealed selective interactions between oxelaidin and RRAD. The mechanism by which butamirate and oxelaidin inhibits GBM cell growth involves the suppression of STAT3 transcriptional activity, leading to down-regulation of cyclin D1 and survivin. In addition, components of RRAD-associated signaling cascades, including p-EGFR, p-Akt, and p-STAT3, were inhibited upon oxelaidin treatment. Intraperitoneal administration of oxelaidin or butamirate markedly suppressed tumor growth in a glioblastoma xenograft mouse model without significant adverse effects. Our collective findings indicate that oxelaidin and butamirate exert anti-tumor effects in glioblastoma, supporting its utility as a novel therapeutic candidate for glioblastoma.

## Introduction

Glioblastoma is an aggressive brain tumor with a survival rate of less than 10%^1^. The majority of patients eventually experience recurrence with the alkylating agent currently used as standard therapy, temozolomide^2^, highlighting the necessity for novel therapeutic strategies against glioblastoma. Drug repositioning may be applied as an effective strategy, provided new indications for an existing drug are identified.

Abnormal activation of receptor tyrosine kinase signaling pathways is frequently observed in glioblastoma, and STAT3 activation is often accompanied by epidermal growth factor receptor (EGFR) overexpression in high-grade gliomas^3^. Activation of STAT3 is essential for the maintenance of cancer stem cell properties in glioblastoma^4^. In the cytoplasm, activated receptors recruit and phosphorylate STAT3 protein at Tyr705. EGFR also serves as a scaffold for trafficking of STAT3 in receptor-ligand complexes from the plasma membrane to the perinuclear region^5,6^.

Previously, we and others showed that RRAD (Ras-related associated with diabetes, RAD) expression is positively correlated with malignant progression^7–10^. RRAD triggers STAT3 activation through physical interactions with the EGFR/STAT3 complex and endosome-mediated nuclear translocation of EGFR^10^. EGFR vIII and RRAD expression in glioblastoma tissue is associated with poor prognosis and correlated with activation of the EGFR/STAT3 pathway along with resistance of cancer cells to cytotoxic drugs^11,12^ .

In view of its pro-tumorigenic role in glioblastoma, RRAD may serve as a promising target for therapeutic intervention. However, RRAD inhibitors are not currently available for clinical purposes. To identify RRAD inhibitors, we used a cell-based assay with a high RRAD-expressing glioblastoma cell line for screening ~ 2000 clinical compounds as a drug repositioning strategy. We focused on compound-induced phenotype changes in live cells grown in three-dimensions as multicellular tumorspheres, with a view to highlighting those that possess inhibitory activity against glioblastoma. Based on screening analyses, five classes of drugs were determined as potent inhibitors of RRAD-expressing glioblastoma, including antitussive agents, anthelmintics (AH), microtubule inhibitors (MI) and topoisomerase inhibitors (TI). However, owing to the nonspecific cytotoxicity (MI/TI) and efficacy (AH) against cancers, we focused on the antitussive agent, oxelaidin, which remained unknown for its anti-cancer activity. Oxelaidin interacted specifically with RRAD in a virtual docking study and effectively suppressed EGFR/STAT3 signaling *in vi*tro. Data from the current study showed that oxelaidin and its derivative butamirate markedly inhibit growth of glioblastoma-derived tumorspheres in vitro and implanted glioblastoma in vivo. Our collective results support the repurposing of oxelaidin as a RRAD-targeting drug for treatment of glioblastoma.

## Results

### Screening clinically used compounds for repositioning as RRAD expressing glioblastoma therapeutics

**Figure 1:**
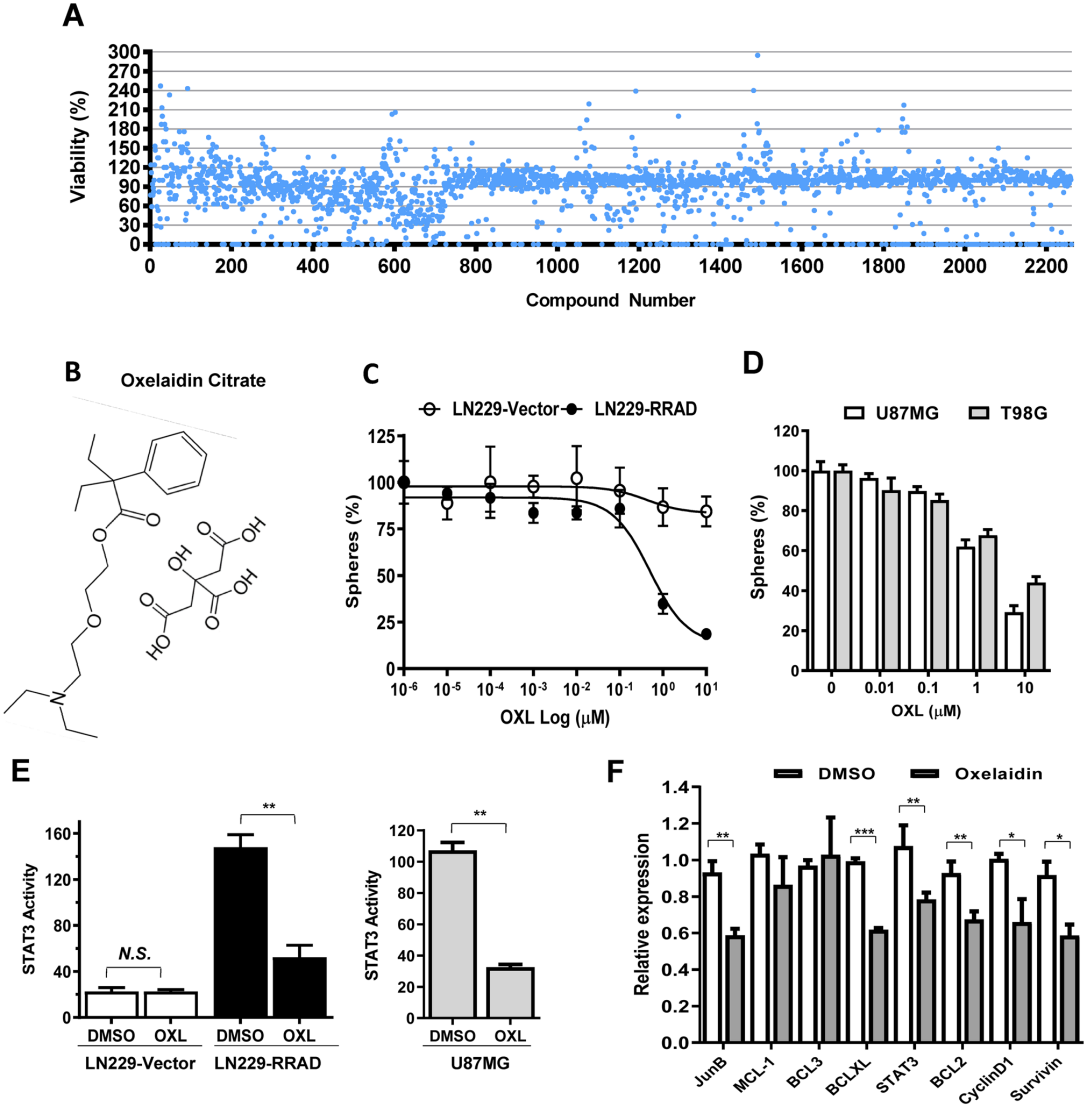
Screening of RRAD inhibitors for drug repositioning using LN229-RRAD human glioblastoma tumorspheres. (**A**) RRAD inhibitor screening was performed using the 2,261 clinical compound library in LN229 cells expressing RRAD (LN229-RRAD). LN229-RRAD cells were treated with 3 μM of each compound for 7 days and the number of tumorspheres counted under a phase-contrast microscope. (**B**) Chemical structure of oxelaidin. (**C**) Dose–response curves showing the survival effect of RRAD overexpression. Cells were treated with the indicated doses of oxelaidin for 7 days, followed by tumorsphere counting. (**D**) Effect of OXL on growth of glioblastoma cells (U87MG and T98G). Cells were treated with OXL for 7 days, followed by tumorsphere counting. (**E**) STAT3 activity in LN229-RRAD and U87MG is downregulated by OXL. A STAT3-dependent luciferase reporter system was established in LN229-Vector, LN229-RRAD, and U87MG. After OXL treatment (5 μM for 2 h), STAT3 activity was monitored using the luciferase assay. (**F**) qRT-PCR assay of endogenous STAT3 target gene expression in LN229-RRAD tumorspheres treated with OXL for 4 days using GAPDH as a loading control. (*P ≤ 0.05; **P ≤ 0.01; ***P ≤ 0.001).

Analysis of gene expression of human glioma tissue samples deposited in the REMBRANDT database revealed a correlation between upregulation of RRAD in EGFR-expressing glioma patients and poorer prognosis^10^. These findings suggest that expression of RRAD is critical for malignant progression of human glioma, supporting the theory that targeting RRAD may prolong the survival of RRAD-overexpressing glioblastoma patients. The aim of this study was to identify clinically used substances which show unforeseen anti-cancer activity against glioblastomas displaying high RRAD expression. To this end, we screened a collection of 2,261 FDA-approved or discontinued compounds from the Korea Chemical Bank. Drug screening led to the isolation of compounds with selective activity towards RRAD-overexpressing glioblastoma cells (LN229-RRAD) in tumorsphere formation assays (Fig. 1A). About 50 hit compounds displayed selective inhibitory effects against LN229-RRAD but were less active in empty vector-transfected cells (Supplementary Fig. 1A,B,C). To validate RRAD-selective activity of these compounds, we performed extensive dose–response experiments using tumorsphere cultures of both LN229-Vector and LN229-RRAD. Multiple candidate compounds were identified by chemical library screening based on sphere formation assay in LN229, U87MG and T98G (Supplementary Fig. 1D). The active compounds identified included antitussive, topoisomerase I inhibitors, microtubule inhibitors and antipsychotic drugs (Supplementary Fig. 2). However, owing to the nonspecific cytotoxicity (MI/TI) and well-known efficacy (AH) against cancers, we focused on the antitussive agent, oxelaidin, which remained unknown for its anti-cancer activity (Fig. 1B). The anti-cancer effects of oxelaidin were investigated using three different glioblastoma cell lines (LN229, U87MG and T98G). In Fig. 1C&D, dose–response curves show that oxelaidin effectively inhibited tumorsphere formation in a dose-dependent manner in RRAD-high (LN229-RRAD, U87MG and T98G) but not RRAD-low glioblastoma cells (LN229-Vector).

**Figure 2:**
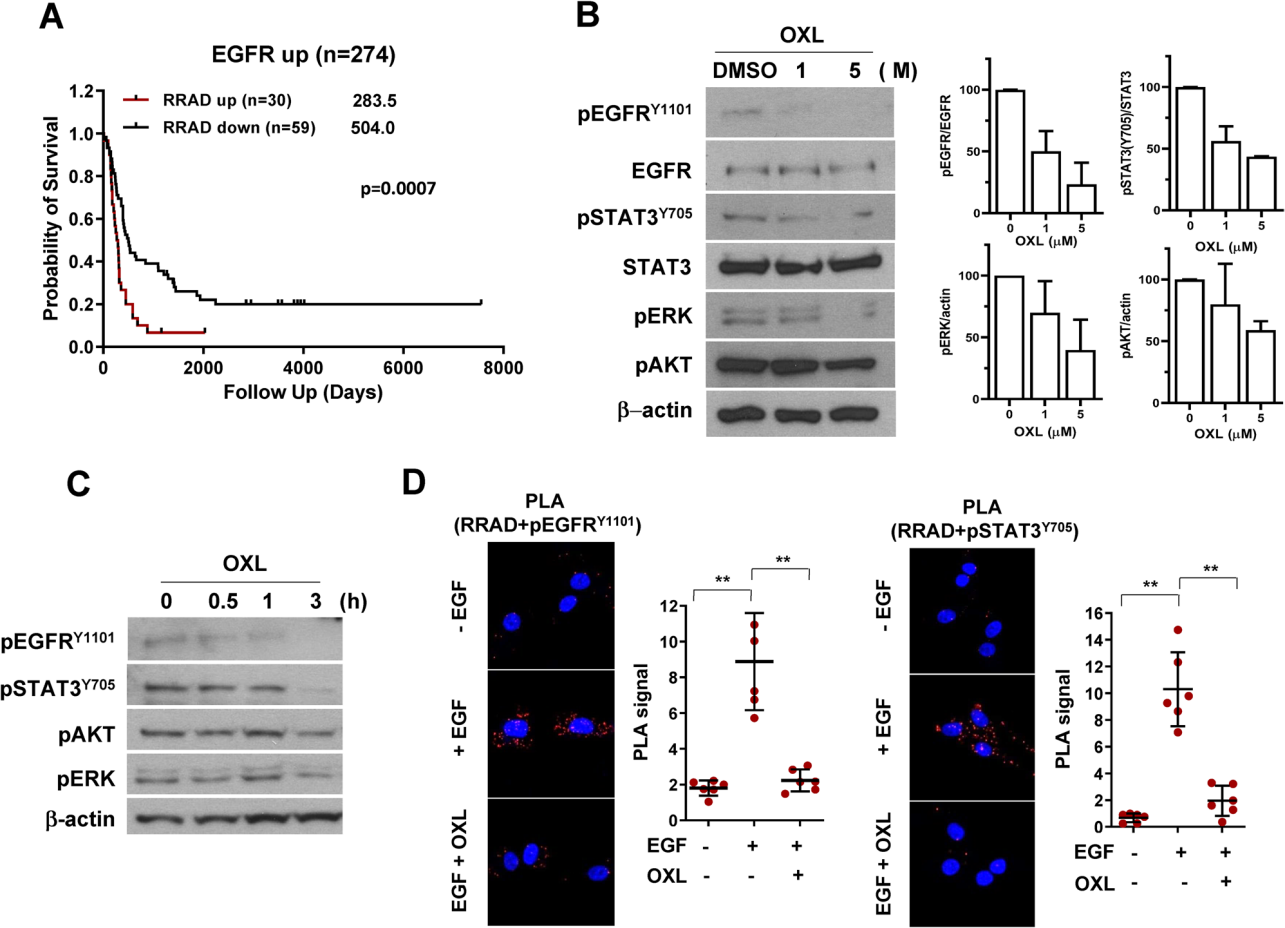
Effects of oxelaidin on EGFR and STAT3 signaling in glioblastoma. (**A**) Kaplan–Meier survival curve for EGFR high patients with GBM according to high (n = 30) and low (n = 57) expression levels of RRAD. All glioma patient data were publicly available in the de-identified form and obtained from the NCI Repository for Molecular Brain Neoplasia Data (REMBRANDT). Data mining and statistical analyses were performed using Project Betastasis software (http://www.betastasis.com/glioma/). (**B**) U87MG tumorspheres were treated with the indicated concentration (0, 1, 5 μM) of OXL for 3 h. (**C**) U87MG tumorspheres were treated with 5 μM of OXL for 0, 0.5, 1 and 3 h. Protein expression of pEGFR, pSTAT3, pAKT and pERK detected via western blot using actin as a loading control. (**D**) PLA of RRAD and activated EGFR or STAT3 performed using RRAD antibodies and either pEGFR Y1101 or pSTAT3 Y705 antibodies in U87MG. Cells were pretreated with OXL for 2 h and subsequently stimulated with EGF (50 ng/mL) for 15 min. Red dots represent interaction signals. Nuclei were stained with the DAPI. Fluorescent signals were detected and images captured at × 400 magnification (Error bars represent ± S.D.; **P ≤ 0.01).

RRAD expression is reported to promote cell survival via activation of the EGFR/STAT3 signaling pathway and stimulate glioblastoma cell migration^10^. To examine whether oxelaidin affects STAT3 transcriptional activity, a STAT3-luciferase reporter construct harboring STAT3 binding sites was transfected into glioblastoma cell lines and luciferase activity measured. As shown in Fig. 1E oxelaidin treatment resulted in significant inhibition of STAT3 activity in U87MG and LN229-RRAD. And qRT-PCR findings additionally showed that multiple endogenous STAT3 target genes including cyclin D1and survivin, many of which are crucial in cancer cell proliferation and survival, were substantially downregulated by oxelaidin (Fig. 1F).

### Oxelaidin inhibits EGFR/STAT3 signaling pathway in RRAD expressing glioblastoma cells

GBM is characterized by abnormal activation of receptor tyrosine kinase signaling pathways, and constitutively activated STAT3 is frequently coexpressed with epidermal growth factor receptor (EGFR) in high-grade gliomas. RRAD expression is reported to activate EGFR/STAT3 signaling pathway^10^. Therefore we further validated RRAD overexpression in EGFR high human glioma specimens. Using the NCI Repository for the Molecular Brain Neoplasia Data (REMBRANDT), we evaluated the survival rates of EGFR high patients according to their RRAD mRNA levels (Fig. 2A). We observed significant differences in terms of survival in High RRAD vs Low RRAD populations (*P* = 0.0007). However, analysis of gene expression in EGFR-low human glioma tissue samples did not reveal a correlation between upregulation of RRAD and poorer prognosis (Supplementary Fig. 3). Accordingly, we focused on whether oxelaidin affects phosphorylation of downstream molecules in EGFR/STAT3 signaling pathways, including p-EGFR, p-AKT, p-ERK, and p-STAT3. Phosphokinase screening of U87MG tumorspheres revealed that oxelaidin suppressed phosphorylation of multiple signaling proteins, including ERK, AKT, and STAT3 (Supplementary Fig. 4). Notably, after treatment of U87MG tumorspheres with oxelaidin, we observed a time- and dose-dependent decrease in pEGFR, pSTAT3 and pERK levels via western blot (Fig. 2B,C). Active STAT3, a transcription factor, translocates to the nucleus to induce transcription of specific target genes and inhibition of its nuclear localization may impede subsequent transcriptional activity. RRAD is reported to interact with EGFR and STAT3 and facilitate translocation of these molecules to the nucleus in glioblastoma^10^. To evaluate the interactions between pEGFR (or pSTAT3) and RRAD in situ, we used a proximity ligation assay (PLA) with antibodies recognizing pEGFR Y1101, pSTAT3 Y705 and RRAD, visible as red fluorescence/PLA signals. EGF treatment promoted interactions of RRAD with pEGFR and pSTAT3, which were suppressed in the presence of oxelaidin (Fig. 2D). Taken together, our results suggest that RRAD-binding oxelaidin inhibits EGF-induced activation of the EGFR/STAT3 signaling pathway.

**Figure 3:**
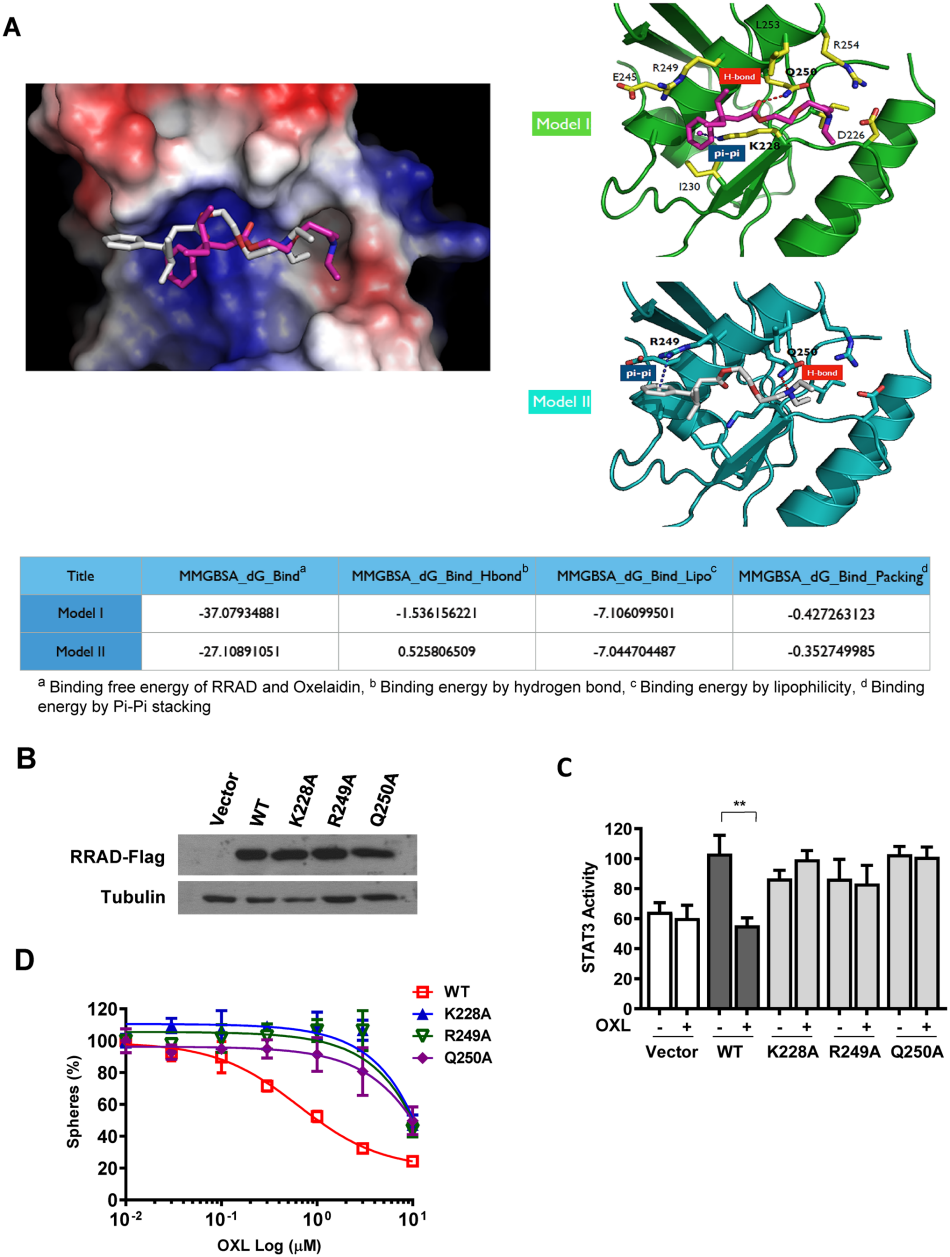
In silico prediction of docking simulation between RRAD and oxelaidin. The molecular docking study on OXL was based on X-ray crystal structures of RRAD (PDB code: 3Q72). (**A**) The pharmacophore features correspond to compound interactions with two binding poses of OXL (white and pink). Red: negative charge, blue: positive charge. Two potent docking simulation models are shown. Hydrogen bond interactions (H-bond) between Q250 (bold) and OXL (pink; model I, white; model II) are represented by a red dotted line. Pi-Pi stacking interactions (pi-pi) between K228 (model I) or R249 (model II) and OXL are represented with a blue dotted line. Energy profile of predicted binding poses obtained through molecular dynamic simulations and MM/GBSA calculations. (**B**) RRAD-FLAG expression was detected via western blot using tubulin as a loading control. LN229 cells were transfected with empty vector, wild-type RRAD or the RRAD mutated at the OXL interaction site (K228, R249, and Q250). (**C**) Mutation of the putative oxelaidin interaction sites of RRAD rescues the inhibitory effect of OXL (5 μM) on STAT3 activity of LN229 glioblastoma cells (**P ≤ 0.01). (D) Dose–response curves detailing the survival effects of mutant RRAD on tumorsphere formation. Cells were treated with the indicated doses of OXL for 7 days, followed by tumorspheres counting.

**Figure 4:**
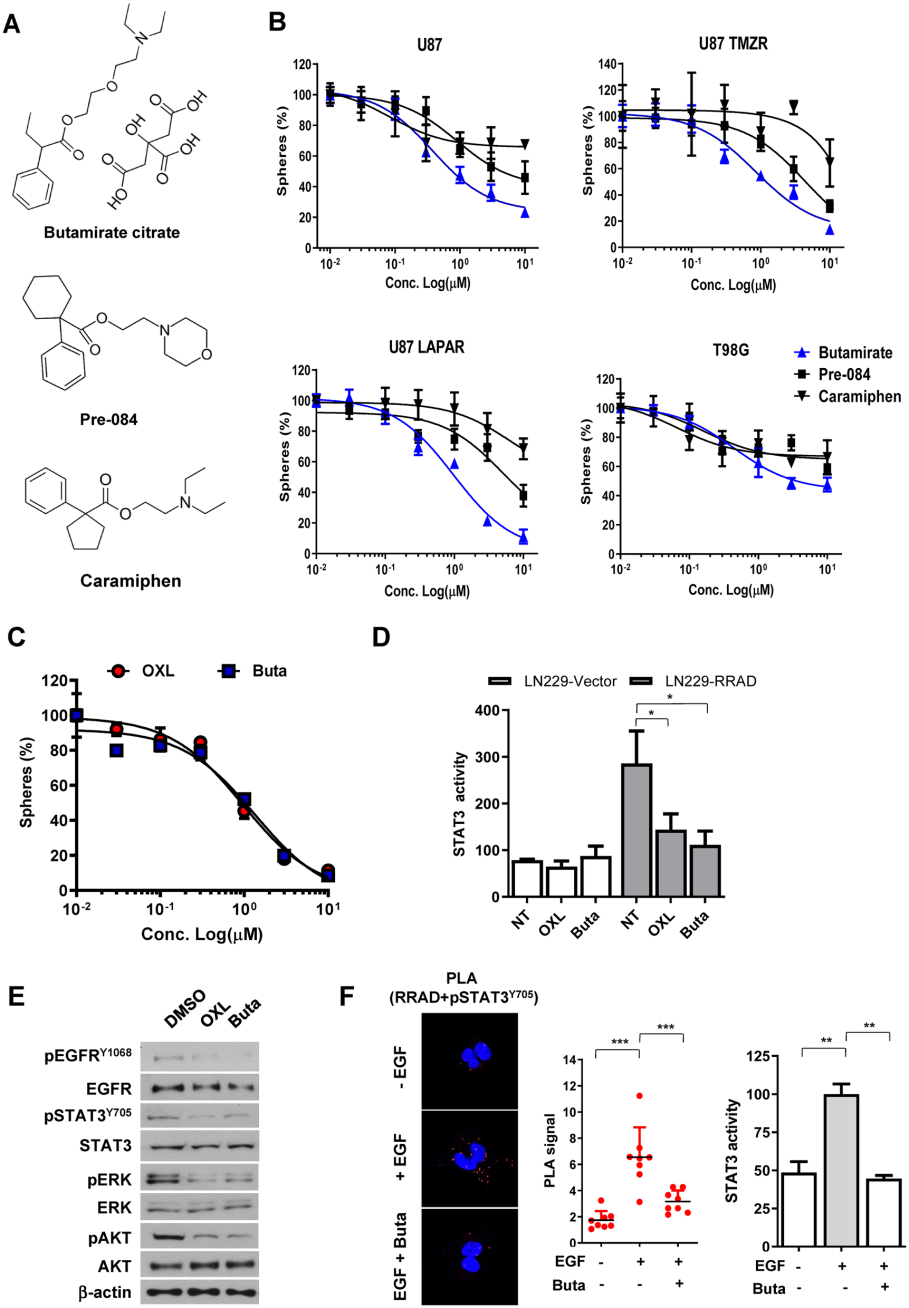

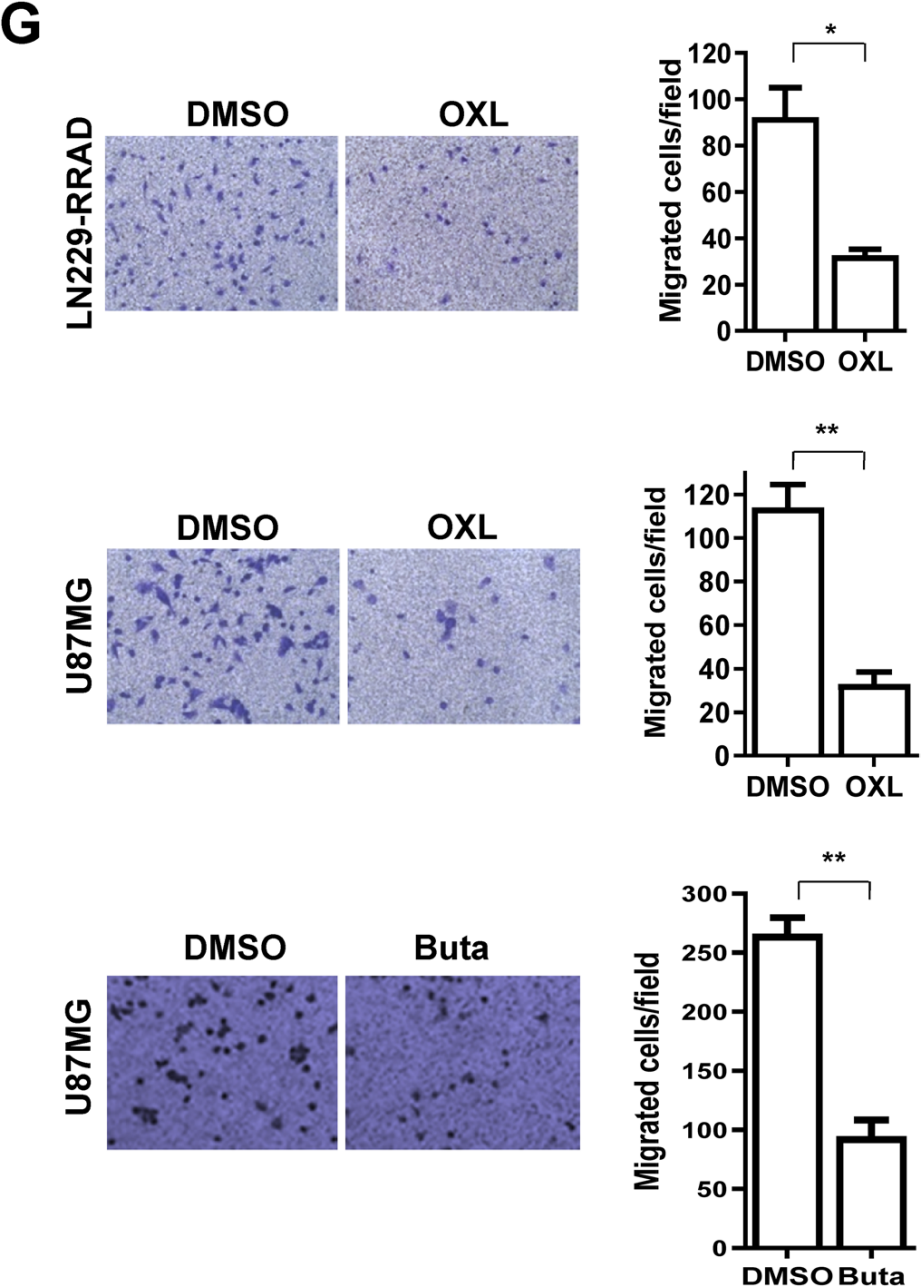
Comparison of the effects of oxelaidin with those of the structurally related antitussive agents. (**A**) Structures of antitussive agents, butamirate citrate, pre-084 and caramiphen. (**B**) U87MG, U87MG-TMZ-resistant (U87 TMZR), U87MG-lapatinib-resistant (U87 LAPAR) and T98G (TMZ resistant) tumorspheres were cultured in the presence of indicated drug for 7 days and the number of tumorspheres counted. (**C**) Dose–response curves showing the survival of LN229-RRAD after treatment with the indicated doses of oxelaidin or butamirate for 7 days, followed by tumorsphere counting. Western blot analysis of protein expression of pEGFR, pSTAT3, pAKT and pERK using actin as a loading control. (**F**) PLA of RRAD and activated STAT3 in U87MG performed using RRAD antibodies and pSTAT3 Y705 antibodies. U87MG cells were pretreated with butamirate citrate for 2 h, followed by stimulation with EGF (50 ng/mL) for 15 min. Red dots represent interaction signals. Nuclei were stained with DAPI. Representative images from analyses of two independent slides. Fluorescent signals were detected and images captured at × 400 magnification (**P ≤ 0.01 and ***P < 0.001). Error bars represent ± SD. (**D**) Butamirate downregulates STAT3 activity in LN229-RRAD but not in LN229-Vector cells. (**E**) Butamirate downregulates pEGFR, pSTAT3, pAKT and pERK Western blot analysis of protein expression of pEGFR, pSTAT3, pAKT and pERK using actin as a loading control. (**F**) Inhibitory effects of Butamirate on EGF-induced STAT3 activity and the RRAD-pSTAT3 interaction. PLA of RRAD and activated STAT3 in U87MG performed using RRAD antibodies and pSTAT3 Y705 antibodies. U87MG cells were pretreated with butamirate for 2 h, followed by stimulation with EGF (50 ng/mL) for 15 min. Red dots represent interaction signals. Nuclei were stained with DAPI. Representative images from analyses of two independent slides. Fluorescent signals were detected and images captured at × 400 magnification. Error bars represent ± SD. (**G**) Transwell migration assay. LN229 and U87MG cells were exposed to 5 μM oxelaidin or butamirate for 2 h before cell migration. Results are presented as means ± SD (n = 3). (*P ≤ 0.05; **P ≤ 0.01; ***P ≤ 0.001).

### Oxelaidin targets RRAD

To investigate whether oxelaidin directly interacts with RRAD protein, binding modes were predicted via molecular docking analyses (New Drug Development Center, Daegu-Gyeongbuk Medical Innovation Foundation, Daegu, Korea). The crystal structure of RRAD was retrieved from the protein data bank (PDB code: 3Q72) and employed in the docking calculations performed with the Glide module in Schrodinger molecular simulation package. For oxelaidin, we selected the binding pose based on a more negative docking score. Docking results suggested two possible binding poses of human RRAD (Fig. 3A). The energy profile and stability of the predicted binding poses were investigated through molecular dynamic simulations and MM/GBSA calculations to obtain the final binding model and key residues. The two predicted binding modes are shown in Fig. 3A. Hydrogen bond interaction (H-bond) between Gln250 and the compound was more stable in model I. Pi-pi stacking interactions between Lys228 (model I) or Arg249 (model II) of RRAD and oxelaidin were similar in both models. However, the binding energies of model I were lower than those of model II.

To establish the specific amino acids critical for oxelaidin binding, we constructed three different binding site mutants (K228A, R249A and Q250A) of the RRAD expression vector and generated stably expressing LN229-RRAD mutant cells. RRAD-mutated stable cell lines displayed similar RRAD expression levels and STAT3 activity as wild-type LN229-RRAD (Fig. 3B,C). However, oxelaidin could not suppress STAT3 activity induced by mutant RRAD (Fig. 3C) or inhibit tumorsphere formation of these cells (Fig. 3D), clearly implying that K228, R249 and Q250 sites of RRAD are essential for interaction with oxelaidin.

### Effects of other antitussive agents on glioblastoma

We further examined the potential anti-glioma effects of other antitussive reagents. The structures of several antitussive compounds are shown in Fig. 4A. Tumorsphere formation data disclosed a comparable anti-glioma efficacy of butamirate which is structurally similar to oxelaidin (Fig. 4B). Previously, we have shown that temozolomide (TMZ) resistance increased in a RRAD expression-dependent manner and RRAD enhanced EGFR protein stability^10^. To demonstrate effects of antitussive compounds on the growth of glioblastoma cell lines, we used U87MG, temozolomide-resistant U87MG-TMZR, lapatinib (EGFR inhibitor)-resistant U87MG-LAPAR, and chemoresistant T98G. Butamirate effectively suppressed growth of glioblastoma cell lines, including U87MG-TMZR and U87MG-LAPAR. As we expected, RRAD expression level was higher in lapatinib-resistant U87MG-LAPAR than U87MG control cells (Supplementary Fig. 5A). Additional expression of RRAD increased resistance to lapatinib, whereas RRAD knockdown increased sensitivity to lapatinib (Supplementary Fig. 5B,C), indicating that RRAD expression plays a critical role in lapatinib resistance as well as TMZ. Butamirate could effectively suppress sphere formation (Fig. 4C) and STAT3 activity as well (Fig. 4D). U87MG treated with oxelaidin or butamirate showed decreased EGFR, STAT3, ERK and AKT phosphorylation, compared with untreated controls (Fig. 4E). Using the proximity ligation assay, we further showed that butamirate effectively inhibits interactions of RRAD with pSTAT3 (Fig. 4F). Examination of the effects on glioblastoma cell migration showed that LN229-RRAD and U87MG cells treated with oxelaidin or butamirate display reduced ability to migrate compared with untreated controls in a transwell migration assay (Fig. 4G).

**Figure 5:**
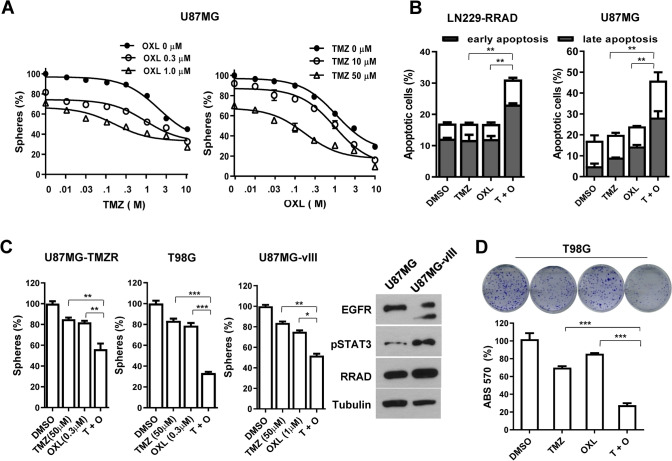
Combination effect of TMZ and OXL on growth and chemoresponse of glioblastoma cell lines. (**A**) Dose–response curves of TMZ in combination with different concentrations of OXL (0, 0.3, 1 μM) for U87MG cells (left) and dose–response curves of OXL in combination with different concentrations of TMZ (0, 10, 50 μM) for U87MG cells (right). (**B**) LN229-RRAD and U87MG tumorspheres were cultured in the presence of TMZ (50 μM), oxelaidin (0.1 μM) or TMZ plus oxelaidin for 2 days. Cells were harvested after 48 h, stained with Annexin V/PI and analyzed via flow cytometry. (**P ≤ 0.01). (**C**) U87MG-TMZ-resistant (U87MG-TMZR), T98G (MGMT-high) and U87MG vIII (constitutively active EGFR) tumorspheres were cultured in the presence of TMZ, OXL or TMZ plus OXL for 7 days and the number of tumorspheres counted. (**D**) T98G cells were treated with 300 μM TMZ, 10 μM OXL or TMZ plus OXL. The clonogenic assay was performed after 2 weeks of treatment. All data represent mean ± S.D of experiments performed in triplicate. (*P ≤ 0.05; **P ≤ 0.01; ***P ≤ 0.001).

### Combination effect of TMZ and OXL on growth, apoptosis, and chemo-response of glioblastoma cell lines

Temozolomide, an alkylating agent, is the current standard-of-care and only chemotherapeutic agent widely used for patients with glioblastoma. However, > 90% recurrent glioblastomas do not respond to repeated challenge with temozolomide. Therefore, we further investigated the combined effects of oxelaidin and temozolomide on U87MG tumorspheres using fixed doses of one drug while titrating the other. The combination of temozolomide and oxelaidin resulted in a significant shift in the inhibition curve, compared with treatment with either drug alone (Fig. 5A). The statistical combination index (CI) was further determined to evaluate whether the combination therapy was synergistic, additive or antagonistic. Drug combination index calculated with CompuSyn software indicated synergistic interactions (Supplementary Fig. 6). Annexin V and PI staining were performed to evaluate the pro-apoptotic activity of oxelaidin in glioblastoma cells. Flow cytometry analysis of stained cells could be effectively used to distinguish dead cells in the early apoptosis (Annexin V-positive/PI-negative) and late apoptosis (Annexin V-positive/PI-positive) groups, showing that oxelaidin combined with temozolomide induces significant increases in both early and late apoptotic populations in LN229-RRAD and U87MG cells (Fig. 5B).

**Figure 6:**
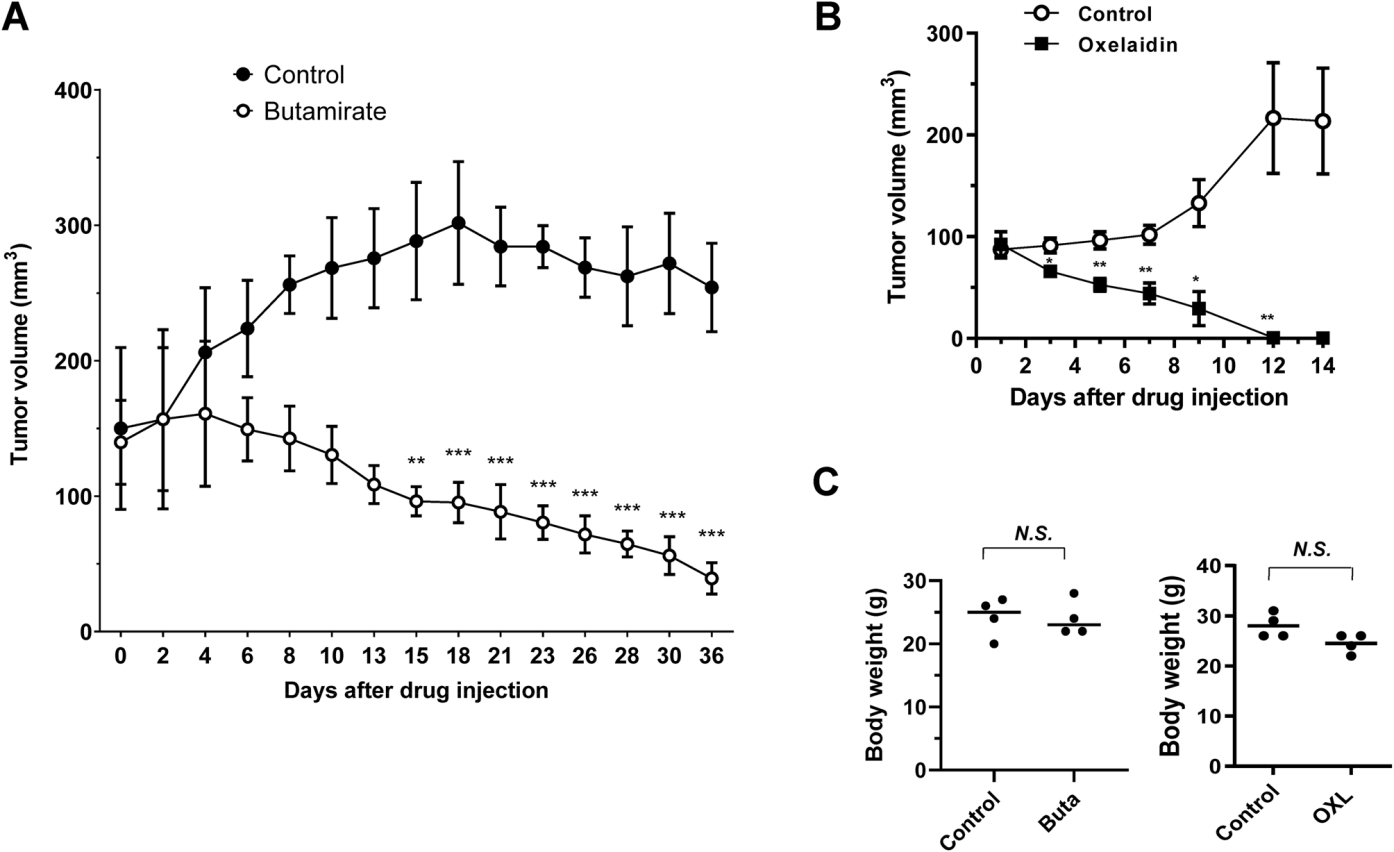
Effects of butamirate and oxelaidin on in vivo tumorigenesis of glioblastoma. (**A**) BALB/c nude mice were subcutaneously injected with U87MG cells and intraperitoneally administered 2 mg/kg butamirate or vehicle control for 36 days. (**B**) BALB/c nude mice were subcutaneously injected with U87MG cells and intraperitoneally administered 130 mg/kg oxelaidin or vehicle control for 14 days (5 days on, 2 days off). Tumor volumes were measured with a caliper every second day. Results are presented as means ± SEM. (**C**) Animal body weights were monitored 36 days (butamirate) or 14 days (oxelaidin) after injection. Lines represent mean values for each group (n = 4; *P < 0.05 and **P < 0.01 vs. vehicle*, t-*test). N.S., not significant.

Amplification of *epidermal growth factor receptor (EGFR)* and its active mutant *EGFR vIII* occurs frequently in glioblastoma and their expression levels are correlated with poor prognosis of glioma patients^12^. MGMT expression is associated with temozolomide resistance in glioblastoma^11^. Therefore we examined whether oxelaidin is effective in inhibiting chemoresistance of glioblastoma cell lines, including constitutively active EGFR vIII-expressing U87MG (U87MG-vIII), temozolomide-resistant U87MG (U87MG-TMZR) and high MGMT-expressing T98G. Depletion of RRAD increased sensitivity to lapatinib in both U87MG and U87MG-vIII cells (Supplementary Fig. 5C). As shown in Fig. 5C, the effects of oxelaidin (0.3 μM or 1 μM) were comparable to those of temozolomide (50 μM). Oxelaidin plus temozolomide (T + O) induced a more marked decrease in the number of tumorspheres. In addition, long-term colony formation was inhibited more significantly by combination treatment (T + O) than either drug alone (Fig. 5D). These findings clearly suggest that oxelaidin exerts potent inhibitory effects on chemoresistant glioblastoma.

### Butamirate and oxelaidin inhibit tumor growth in a glioblastoma mouse xenograft model

Based on the anti-glioma effect of oxelaidin and butamirate observed in vitro, we examined whether oxelaidin or butamirate inhibits glioblastoma growth in a U87MG xenograft mouse model. Following establishment of U87MG tumor xenografts, mice were injected intraperitoneally with oxelaidin or butamirate and tumor growth inhibition assessed. Tumors in the control group grew rapidly whereas oxelaidin or butamirate-exposed tumors regressed soon after treatment (Fig. 6A,B). Systemic toxicity was evaluated by measurement of body weight (Fig. 6C). No significant differences in body weight were observed in animals treated with either oxelaidin or butamirate relative to control animals, indicating that these drugs exert tolerable toxicity to mice at the curative dose.

## Discussion

High expression of RRAD is clearly implicated in the malignant progression of human glioblastoma. In this study, we have shown that oxelaidin, an antitussive agent, effectively inhibits glioblastoma through targeting RRAD. Growth factors and their receptor tyrosine kinases are highly expressed in glioblastoma and promote tumor progression. EGFR amplification occurs in approximately 40–50% glioblastomas, which results in an increase in prosurvival and proliferative signals. Constitutively activated STAT3 is frequently coexpressed with EGFR in high-grade gliomas^3^ and cooperates with EGFR to facilitate epithelial-mesenchymal transition in human epithelial cancers^13^. In view of our previous finding that RRAD induces EGFR-STAT3 activation and increases chemoresistance of glioblastoma^10^, it may present a promising target for therapeutic interventions. To explore potential RRAD-targeting drugs, we screened an FDA-approved drug library, which led to the identification of multiple drugs including the antitussive agent oxelaidin. Virtual screening analyses further revealed that oxelaidin might interact with RRAD.

In this study, the binding of oxelaidin to RRAD was investigated by both in vitro and in silico experiments. Because the crystal structures of human RRAD have been determined (PDB code: 3Q72), the molecular docking of oxelaidin when complexed with RRAD was performed to determine the position of oxelaidin in the three-dimensional structure of RRAD and the structural details of the interaction sites, with the results confirmed by the lack of oxelaidin-mediated inhibition of STAT3 activation. To date, no inhibitor of RRAD has been identified, and, to our knowledge, the structural mechanism by which oxelaidin inhibits RRAD has not been determined. The in vitro and in silico data presented in this study demonstrate that RRAD inhibition results from the hydrogen bonding between oxelaidin and the Gln250 of RRAD, and the pi-pi stacking interactions of oxelaidin with the Lys228 or Arg249 of RRAD.

Tumorigenicity is preferentially augmented in glioblastoma via activation of STAT3 whereas EGFR contributes to survival of tumor cells. Given the ability of RRAD to stimulate the EGFR/STAT3 pathway, we investigated the influence of oxelaidin on EGFR/STAT3 signaling. Notably, levels of pEGFR, pAKT, pERK, and pSTAT3 were inhibited in the presence of oxelaidin. Data from PLA demonstrated that oxelaidin suppresses interactions between RRAD and EGFR or STAT3 in glioblastoma cells, supporting its utility as an active anti-glioma agent. Consistent with these results, oxelaidin exerted significant inhibitory effects on tumorsphere formation, long-term colony formation, migration, and tumor growth in vivo. Butamirate crosses the blood–brain barrier and binds to the medulla oblongata^14^. However, the in vivo experiments were performed in a subcutaneous xenograft mouse GBM model, which was immunodepressed and does not accurately reflect the complexity of GBM biology. Further experiments using an orthotopic immunocompetent model may help determine whether these antitussive agents are able to cross the BBB targeting the GBM within the CNS, and whether the immune response is involved in the therapeutic efficacy of this approach.

RRAD is a Ras-related GTPase that lacks prenylation motifs and differs from other RAS-related GTPases in a GTP-binding domain and NH2- and COOH-terminal extensions^15^. Ras signaling activates RAF, MEK and ERK along with other proteins, providing a strong rationale for the use of the RAS pathway to develop therapeutic interventions. In malignant gliomas, however, somatic mutations of RAS or RAF are very rare^16–18^. Primary glioblastoma tumors are reported to express significantly low levels of RAS transcripts and no detectable levels of RAS proteins^19^.

To our knowledge, this is the first study to show that oxelaidin or butamirate is capable of modulating EGFR-STAT3 signaling, thereby decreasing temozolomide resistance of glioblastoma cells. Targeting of molecules responsible for temozolomide resistance is crucial to block glioblastoma evasion of therapeutic responses. Temozolomide resistance is associated with increased expression of p53^20,21^, EGFR^22^, STAT3^23^, O6-methylguanine methyltransferase (MGMT) ^11,24,25^, APNG^26^, base excision repair genes^27^, mismatch repair protein^28^, histone demethylase^29^, nicotinamide phosphoribosyl transferase^30^, CD133^31^ and anti-apoptotic genes^32^. These data indicate that temozolomide resistance results from complex cellular events^33,34^. Overexpression of RRAD is associated with poor prognosis of glioblastoma^10^. Given that RRAD induces EGFR and STAT3 activation and RRAD knockdown sensitizes chemoresistant cancer cells to cytotoxic drugs^8,9^, targeting STAT3 with oxelaidin or butamirate may attenuate temozolomide resistance and glioblastoma recurrence after treatment.

## Material and methods

### Chemicals and reagents

Oxelaidin citrate (oxelaidin) was purchased from ApexBio (Hsinchu city, Taiwan), butamirate citrate from Santa Cruz Biotechnology (Dallas, TX), pre-084 from Tocris (Bristol, UK), and Caramiphen HCl from Sigma-Aldrich (St. Louis, MO). All compounds were dissolved in DMSO before use. Temozolomide was obtained from Sigma-Aldrich. STAT3 promoter plasmid (4 × M67 pTATA TK-Luc, #8688) was acquired from Addgene (Cambridge, MA) and pRL-TK renilla luciferase plasmid from Promega (Madison, WI). MSCV-XZ066 EGFR vIII (#20,737) was acquired from Addgene (Cambridge, MA) and cloned into pCMV-Tag4C. Antibodies against RRAD (Abcam, Cambridge, MA), p-ERK (Santa Cruz), AKT, EGFR, p-STAT3 S727 and p-STAT3 Y705, p-EGFR Y1101 and p-EGFR Y1068 (Cell Signaling, Beverly, MA) were used. Horseradish peroxidase-conjugated secondary antibodies were obtained from Santa Cruz. Reactive proteins were visualized using the Thermo-fisher ECL kit (Rockford, IL). The 21-nucleotide-long siRNAs corresponding to RRAD were used as described previously^10^. The plasmid pCMV-Tag2B flag-RRAD wild-type and the mutant constructs K228A, R249A, and Q250A were generated as described^10^. Site-directed mutagenesis was performed according to the manufacturer´s instructions using the plasmid pCMV-Tag2B-RRAD as the template to generate mutations in the oxelaidin binding site (RRAD mutants K228, R249 and Q250 (Q5 site-directed mutagenesis kit, New England Biolabs). Constructs expressing RRAD and binding site mutants were verified by sequencing.

### Cell culture

Human glioblastoma cell lines (LN229, U87-MG, and T98G) were purchased from the American Type Culture Collection (Rockville, MD). Adherent cells were maintained in DMEM with heat-inactivated 10% fetal bovine serum, penicillin, and streptomycin (Gibco BRL, Grand Island, NY). Tumorspheres were cultured in serum-free DMEM/F12 (Invitrogen, Carlsbad, CA) supplemented with basic fibroblast growth factor (20 ng/mL; Invitrogen), EGF (20 ng/mL; BD Biosciences, San Jose, CA) and N2 supplement (1X; Invitrogen). Short tandem repeat (STR) polymorphism analysis of cells was used to confirm cell identity. Cells were maintained in an incubator at 37ºC under 5% CO_2_. To establish stable cell lines overexpressing EGFR vIII and RRAD wild type and mutants, LN229 cells were transfected with control vector or vectors containing EGFR vIII or flag RRAD constructs. Successfully transfected cells were selected with 800 μg/ml of G418, and the expression of EGFR vIII or RRAD confirmed by immunoblotting. U87MG-TMZR cell lines were prepared as described previously^10^. To select GBM cells resistant to lapatinib, U87MG cells were exposed to progressively increasing concentrations of lapatinib (termed U87MG-LAPAR) for 6 months.

### Molecular docking experiment

In silico prediction of docking simulations was employed to analyze direct interactions between RRAD and oxelaidin. Molecular docking studies on oxelaidin were performed based on the X-ray crystal structures of RRAD (PDB code: 3Q72). The protein structure was minimized using the Protein Preparation Wizard in the Maestro graphical user interface (version 9.3, Schrödinger). Oxelaidin was docked onto a minimized crystal structure of RRAD using the docking routine in Prime module. Docking was performed using the default settings and fixed residues. Graphics for the refined docking model for oxelaidin were generated using PyMol (http://www.pymol.org).

### Tumorsphere formation assay

Cells were suspended in DMEM/F12 containing 20 ng/mL EGF, bFGF and N2 supplement (1X) as stem cell permissive medium. Cells were seeded in ultra-low attachment 96 well plate and compounds of the chemical library (Korea Chemical Bank) treated as indicated concentration. Spheres were collected after 7 days and protein extracted for additional experiments or dissociated with Accutase (Invitrogen). To determine the cytotoxic effects on tumorspheres, parallel studies were performed on cells treated with temozolomide (0–50 µM) plus oxelaidin citrate (0–10 µM). The combination index (CI) value was analyzed using CompuSyn software (Chou and Martin, CompuSyn Inc, USA) and calculated with the Chou-Talalay method. CI values < 0.9, 0.9–1.1, and > 1.0 indicate synergistic interactions (more than additive), summation (additive), and antagonistic interactions (less than additive), respectively.

### STAT3 luciferase reporter assay

To examine STAT3-mediated transcriptional activity, cells were seeded in 24-well plates and co-transfected with a STAT3-luciferase reporter construct (4xM67 pTATA TK-Luc) plus *Renilla* luciferase expression vector, pRL-TK (Promega), using Effectene (Qiagen, Hilden, Germany). At 24 h post-transfection, cells were treated with oxelaidin citrate or DMSO for another 2 h. Next, cells were harvested for the luciferase reporter assay performed using the dual-luciferase reporter assay system (Promega). Firefly luciferase values were normalized to those of *Renilla* luciferase.

### Western blot analysis

Whole cell lysates were prepared using ice-cold RIPA buffer (0.5% sodium deoxycholate, 1% Nonidet P-40, 150 mM NaCl, 50 mM Tris [pH 7.5], 0.1% sodium dodecyl sulfate [SDS] and 1 mM phenylmethylsulfonyl fluoride [PMSF]) and cleared via centrifugation (13,000 rpm for 30 min). The protein concentration in each sample was estimated with the BCA assay. Equal amounts of protein were separated via 12% SDS-PAGE and transferred to PVDF membrane. After 1 h of incubation in blocking solution (5% skimmed milk), membrane was cut prior to hybridisation and incubated overnight with primary antibody at 4 °C, followed by horseradish peroxidase-conjugated secondary antibody for 1 h at room temperature. Immunoreactive bands were visualized using enhanced chemiluminescence (ECL) reagent (Amersham Pharmacia Biotech, Arlington Heights, IL).

### Reverse transcription polymerase chain reaction (RT-PCR)

Total RNA was isolated using TRIzol (Invitrogen) and reverse-transcribed using M-MLV reverse transcriptase (Promega) and oligo dT primers according to the manufacturers’ protocol. To analyze relative gene expression, cDNA was subjected to TaqMan-based real-time quantitative PCR on a 7500 Real-Time PCR system (Applied Biosystems, Foster City, CA, USA). Briefly, cDNA for each gene was added to 20 μl PCR mix containing 1 × TaqMan Gene Expression Master Mix (Applied Biosystems). Amplification was carried out under the following conditions: 1 cycle of denaturation (95 °C per 10 min) followed by 40 cycles of two-stage PCR (95 °C for 15 s and 60 °C for 1 min). Fluorescence signals were measured continuously during the repetitive cycles. Data were normalized to GAPDH and the differences in fold change calculated based on the 2^-ΔΔCt^ method.

### Proximity ligation assay (PLA)

PLA was performed using Duolink II Detection Reagent Red (Duolink in situ PLA, Sigma-Aldrich) according to the manufacturer’s protocol. Cells were seeded on a slide and incubated for a further 24 h prior to fixation and permeabilization in 0.1% Triton X-100 in PBS. Non-specific binding was blocked by incubation with Blocking solution (Duolink) for 1 h at RT. Mouse monoclonal antibody targeting pEGFR Y1101 or pSTAT3 Y705 was incubated overnight at 4 °C together with rabbit antibody targeting RRAD. PLA minus probe against rabbit and plus probe against mouse were used in conjunction with the Duolink In Situ PLA Red Kit (Duolink, Sigma-Aldrich) according to the manufacturer’s instructions. PLA signals (red), indicative of protein–protein interaction sites, were observed and images captured using confocal microscopy (Zeiss, LSM 780) at × 400 magnification.

### Transwell migration Assay

Cells (1 × 10^5^) were loaded into the upper chamber of Transwell plates (8 µm pore size) and fetal bovine serum (10%) used as a chemoattractant in the lower chamber. After incubation for 24 h, cells in the lower chamber were fixed and stained with 0.005% (w/v) crystal violet. The number of migrated cells was quantified by counting in five random fields of each membrane.

### Clonogenic assay

Cells were seeded in six-well plates (500 cells/well) and exposed to temozolomide (300 µM) and oxelaidin citrate (10 µM) for 2 h, followed by incubation for 10 to 14 days. Cell densities or colonies were assessed after crystal violet staining. Colonies of more than 50 cells were counted. The dye was subsequently extracted with 10% acetic acid and absorbance determined spectrophotometrically at 570 nm.

### Annexin V and PI staining

Annexin V and PI staining were conducted using an Annexin V-FITC Apoptosis Detection Kit I (BD Pharmingen™, San Diego, CA). Cells were treated with oxelaidin citrate or temozolomide at the indicated concentrations, collected and adjusted to a density of 1 × 10^5^cells/mL. Apoptotic cells were detected via fluorescent staining. Briefly, collected cells were incubated with Annexin V-FITC and propidium iodide (PI) in binding buffer for 15 min in the dark and stained cells immediately subjected to flow cytometry analysis using a FACS verse flow cytometer (BD Biosciences, San Jose, CA).

### Mouse xenograft model

Animal care and all animal-related experimental procedures were conducted in accordance with the approval and guidelines of the Institutional Animal Care and Committee of Samsung Biomedical Research Institute. U87MG human glioblastoma cells were subcutaneously implanted in matrigel in 6- to 8 week-old male BALB/c nude mice (Orient Bio, Korea) and tumor growth monitored using calipers. When tumor sizes reached ~ 50–100 mm^3^, mice were randomly divided into two groups (4 mice per group). The control group was treated with vehicle and the group with butamirate (2 mg/kg) or oxalaidin (130 mg/kg) intraperitoneally for 36 days and 14 days, respectively. Tumor sizes were measured three times per week. Tumor volumes were calculated using the formula: (a × b^2^) × 0.5, in which a is the long axis and b the short axis in mm.

### Patient datasets and data analysis

All glioma patient data were publicly available in the de-identified form and obtained from the NCI Repository for Molecular Brain Neoplasia Data (REMBRANDT). Data mining and statistical analyses were performed using Project Betastasis software (http://www.betastasis.com/glioma/). Significant results yielded using Kaplan–Meier survival analysis are presented as log-ranked *P* values for significance of differences in survival between groups.

### Statistical analysis

All data are presented as mean ± SD of at least three independent experiments. Statistical analyses were performed using analysis of variance (ANOVA) and unpaired Student’s *t*-test, with *P*-values < 0.05 considered significant.

## Supplementary Information

Supplementary Information 1.

Supplementary Information 2.
