# Supplementary material for: Application of the antitussive agents oxelaidin and butamirate as anti-glioma agents
Source: Sci Rep. 2021 May 12;11:10145. doi: 10.1038/s41598-021-89238-9 (PMC8115262; doi:10.1038/s41598-021-89238-9)

Application of the antitussive agents oxelaidin and butamirate as anti-glioma agents

Sook-Ja Lee[1,4], Seon-Yong Yeom[1,4], Jee-Young Lee[3], and Chaehwa Park[1,2*]

Authors' Affiliations:

[1] Research Institute for Future Medicine, Samsung Medical Center, Seoul, Korea

[2] Department of Medicine, Samsung Medical Center, Sungkyunkwan University School of Medicine, Seoul, Korea

[3] New Drug Development Center, Daegu-Gyeongbuk Medical Innovation Foundation, Daegu, Korea

[4] These authors contributed equally to this work.

Corresponding Author: Chaehwa Park, Research Institute for Future Medicine, Samsung Medical Center,

Sungkyunkwan University School of Medicine, Irwon-dong, Seoul, 06351, Korea.

Phone: 82-2-3410-3458, E-mail: cpark@skku.edu

Supplementary Figure 1.

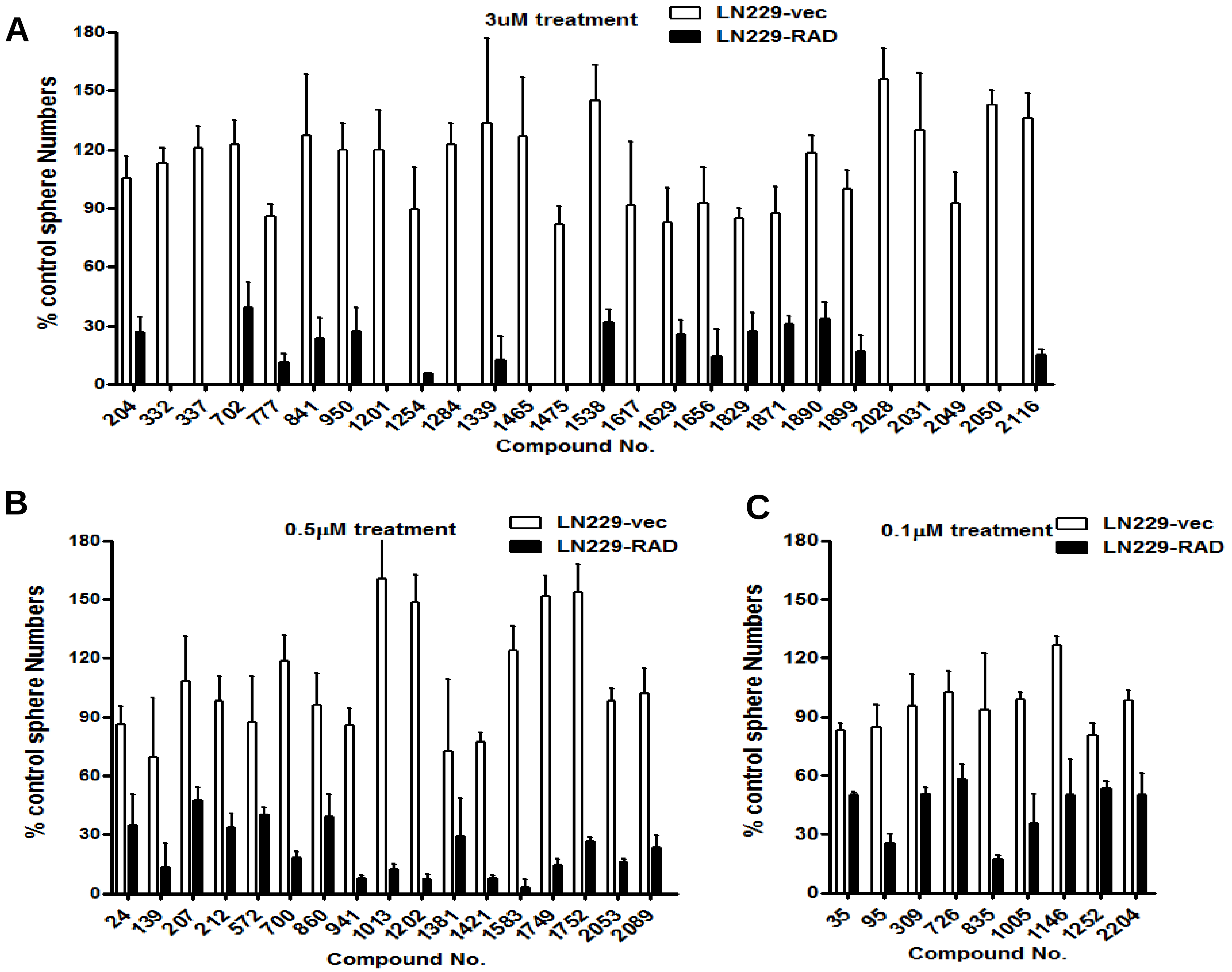

D

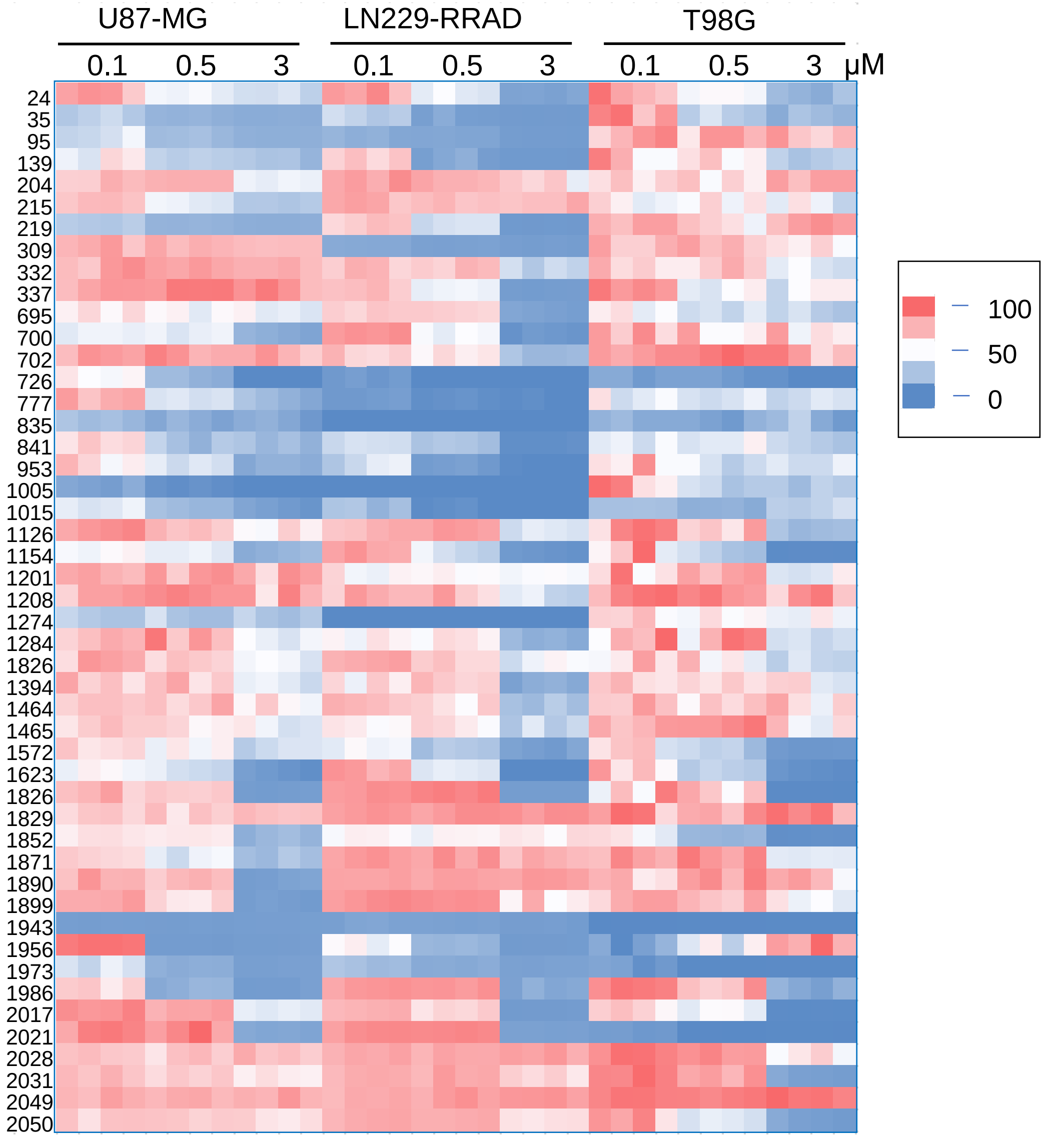

Supplementary Figure 1. Several candidate compounds were identified by chemical library screening based on sphere formation assay for 7 days with 3μmol/L (A), 0.5μmol/L (B), and 0.1μmol/L (C). (D) Effects of selected compounds on three GBM cell lines. Growth inhibition levels were presented with heatmap (% of control).

Supplementary Figure 2.

		IC_{50} (μmol/L)	
**Function**	**Compound**	**LN229-Vector**	**LN229-RRAD**
**Antitussive**	Oxelaidin citrate	*ND	0.495
**Antipsychotic**	Benzotropine mesylate	ND	3.301
	Fluphenazine hydrochloride	ND	0.300
	Pimozide	ND	4.930
	Ritanserin	ND	0.003
**Anthelmintic**	Fenbendazole	1.952	0.272
	Albendazole	ND	0.426
	Parbendazole	0.253	0.031
	Mebendazole	0.603	0.181
**Microtubule inhibitor**	Paclitaxel	0.074	0.005
**Topoisomerase inhibitor**	Topotecan hydrochloride	1.459	0.034
	10-hydroxycamptothecin	0.248	0.004

*ND : Not determined

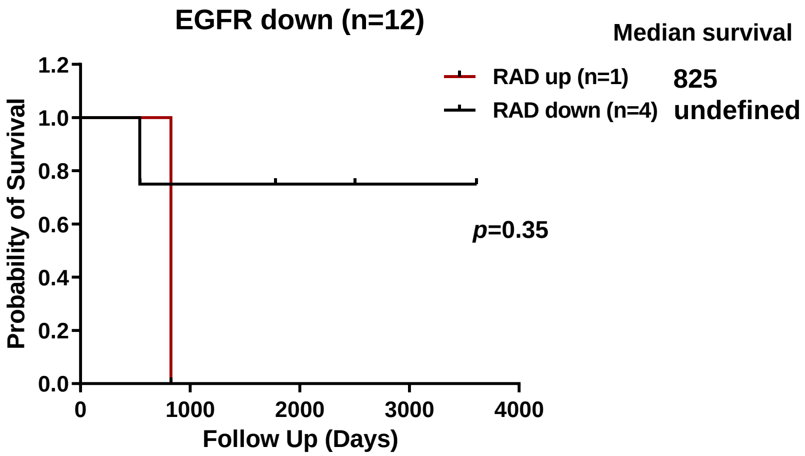

Supplementary Figure 3. Kaplan-Meier survival curves of EGFR-low patients (n=12) with GBM and high (n=1) or low (n=4) levels of RRAD expression. Data on all glioma patients are publicly available in de-identified form in the NCI Repository for Molecular Brain Neoplasia Data (REMBRANDT). Data mining and statistical analyses were performed using Project Betastasis software (http://www.betastasis.com/glioma/).

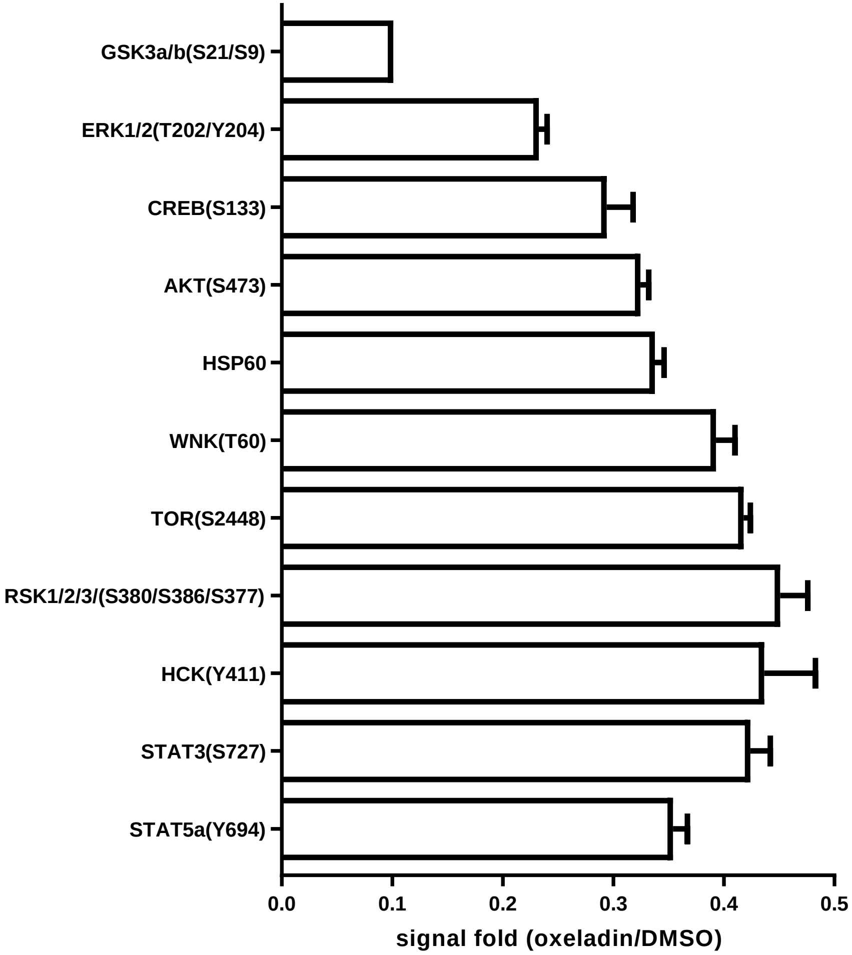

Supplementary Figure 4. Phospho-kinase antibody array performed on protein lysates from U87MG tumorspheres either DMSO or OXL treated for 48h. Cell lysates were hybridized to membranes containing capture antibodies specific for phosphorylated kinases. Signal intensity was quantified using computerized Image J imaging analysis software (NIH).

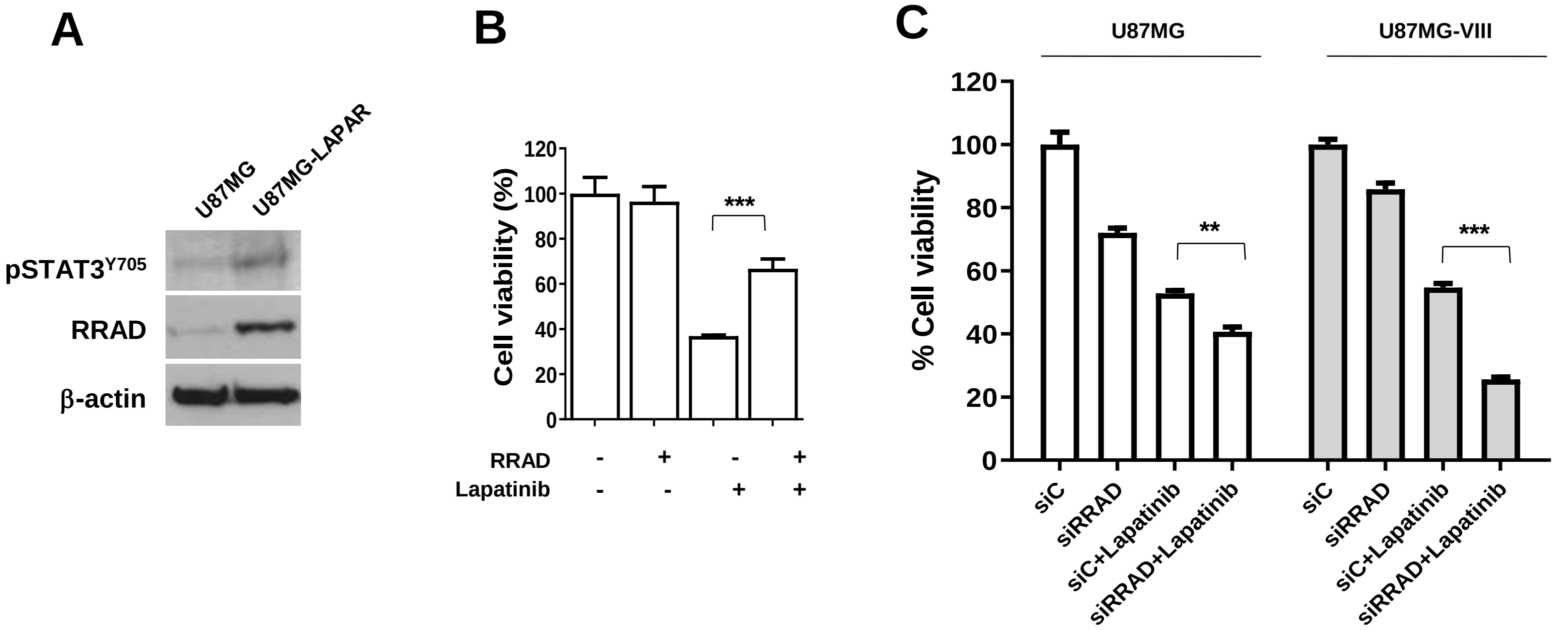

Supplementary Figure 5. GBM cell viability according to RRAD over-expression. (A) Lapatinib-resistant U87MG-LAPAR cells have increased STAT3 phosphorylation and RRAD expression. Cell viability according to RRAD over-expression in LN229 (B) and depletion in U87MG (C) upon lapatinib treatment. (**$P \leq 0.01$; *** $P \leq 0.001$).

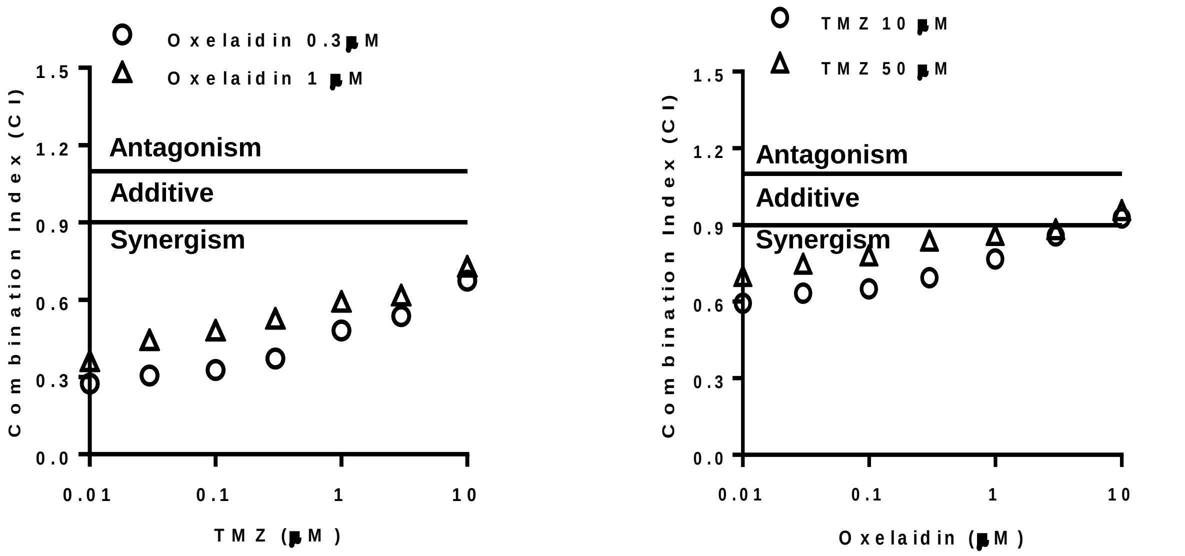

Supplementary Figure 6. oxelaidin improves the efficiency of chemotherapy *in vitro.* Drug combination index were calculated with CompuSyn software.

unedited

Figure 2.

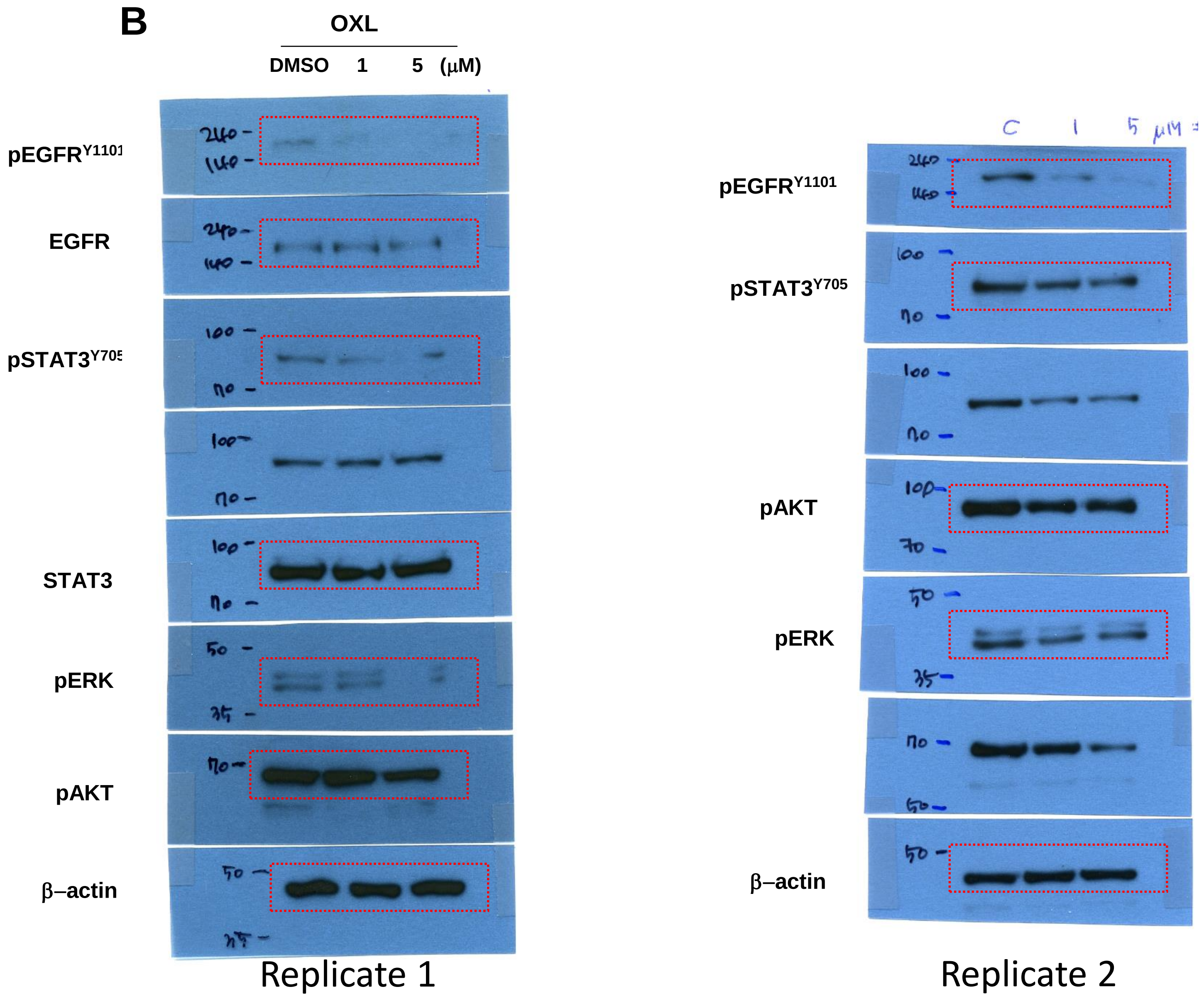

unedited

Figure 2.

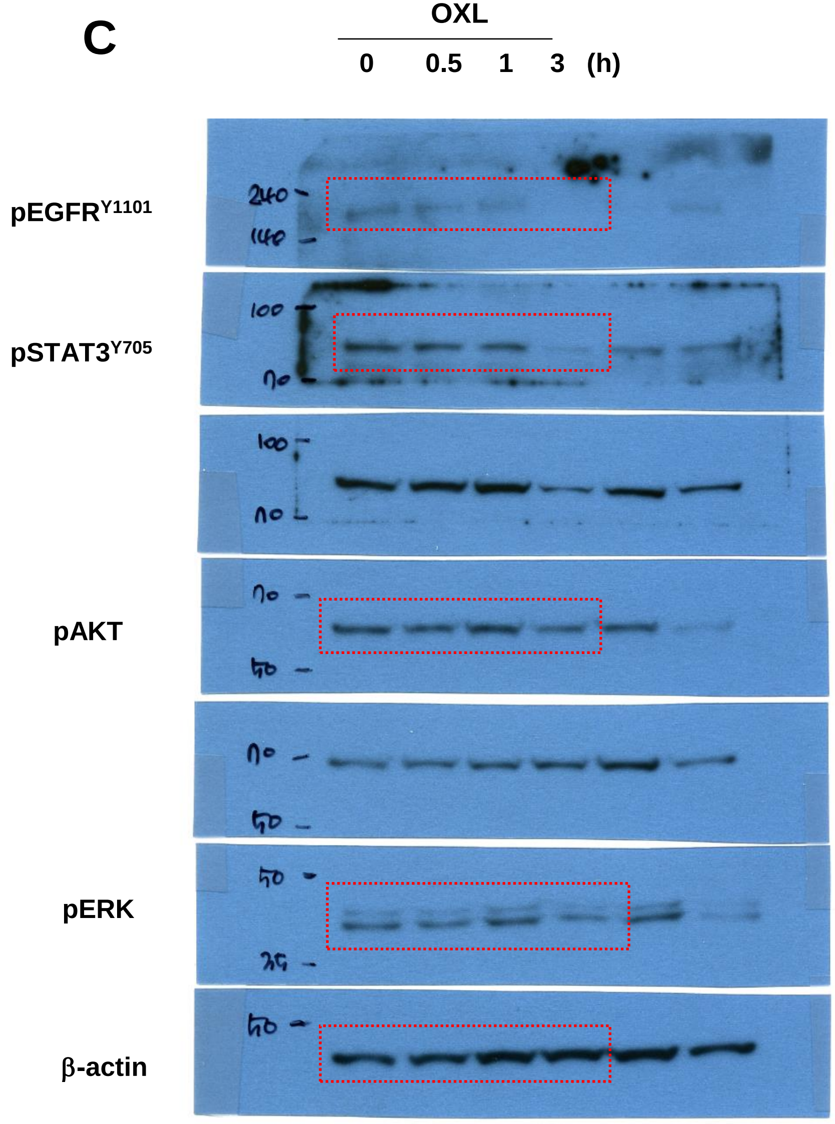

unedited

Figure 3.

B

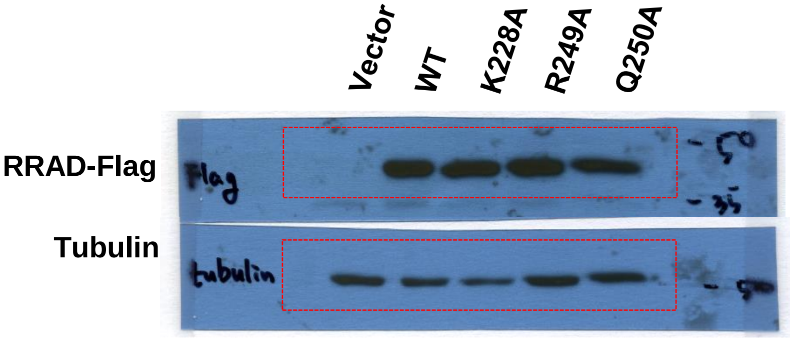

Figure 4.

E

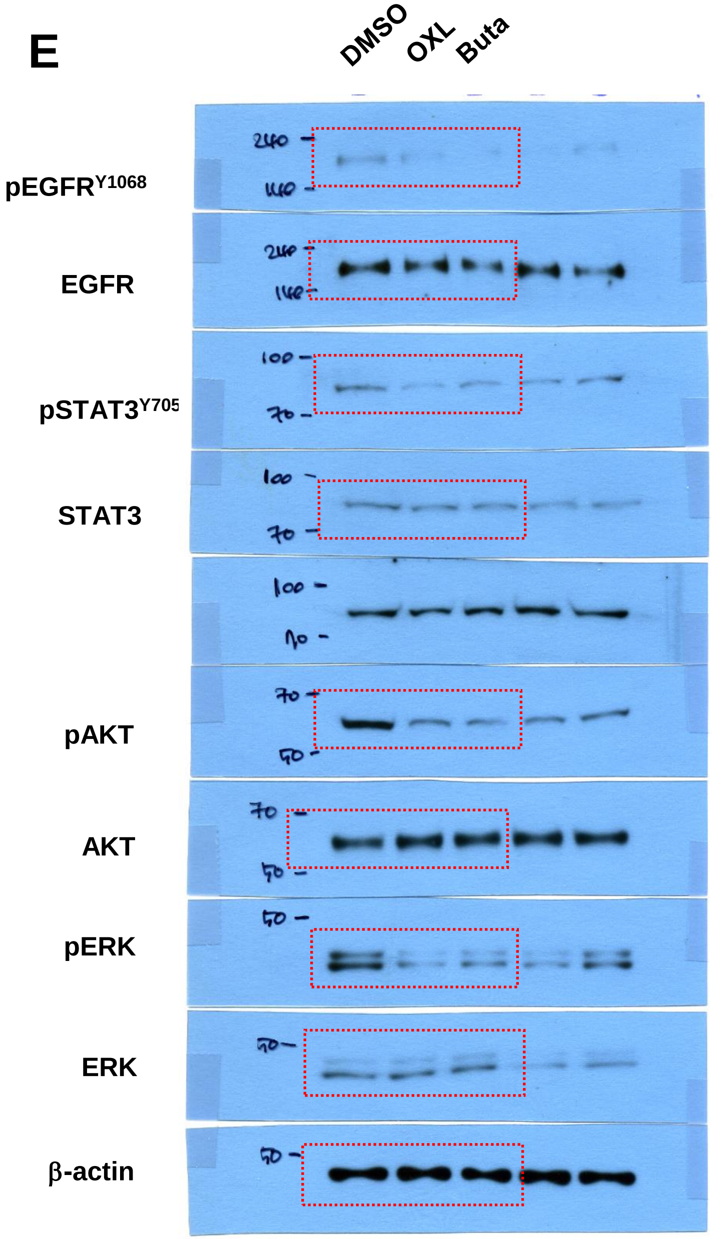

unedited

Figure 5.

Supplementary Figure 5.

C

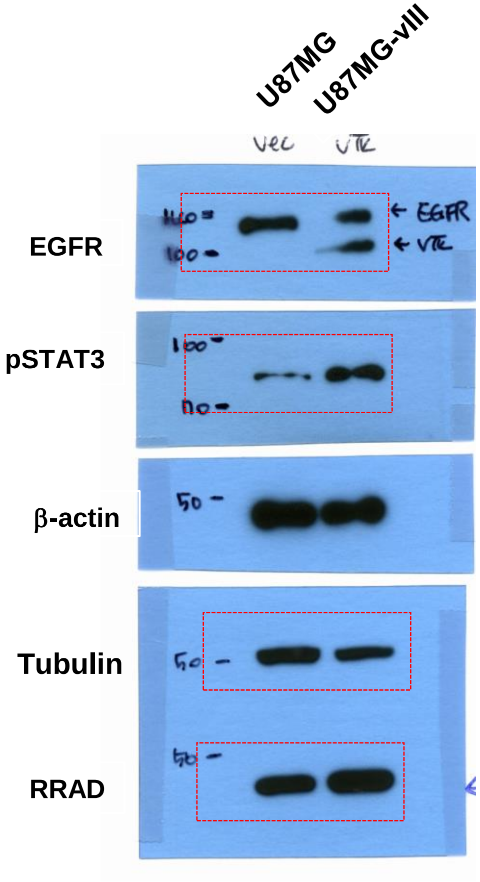

A

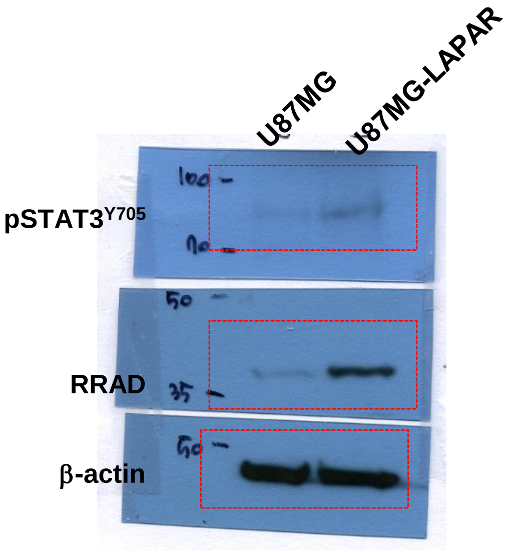

Supplement: Supplementary file 1 — Supplementary Information 1. [file 41598_2021_89238_MOESM1_ESM.pdf]
