# Supplementary material for: Application of the antitussive agents oxelaidin and butamirate as anti-glioma agents
Source: Sci Rep. 2021 May 12;11:10145. doi: 10.1038/s41598-021-89238-9 (PMC8115262; doi:10.1038/s41598-021-89238-9)
Supplement: Supplementary file 2 — Supplementary Information 2. [file 41598_2021_89238_MOESM2_ESM.pptx]

## Slide 1
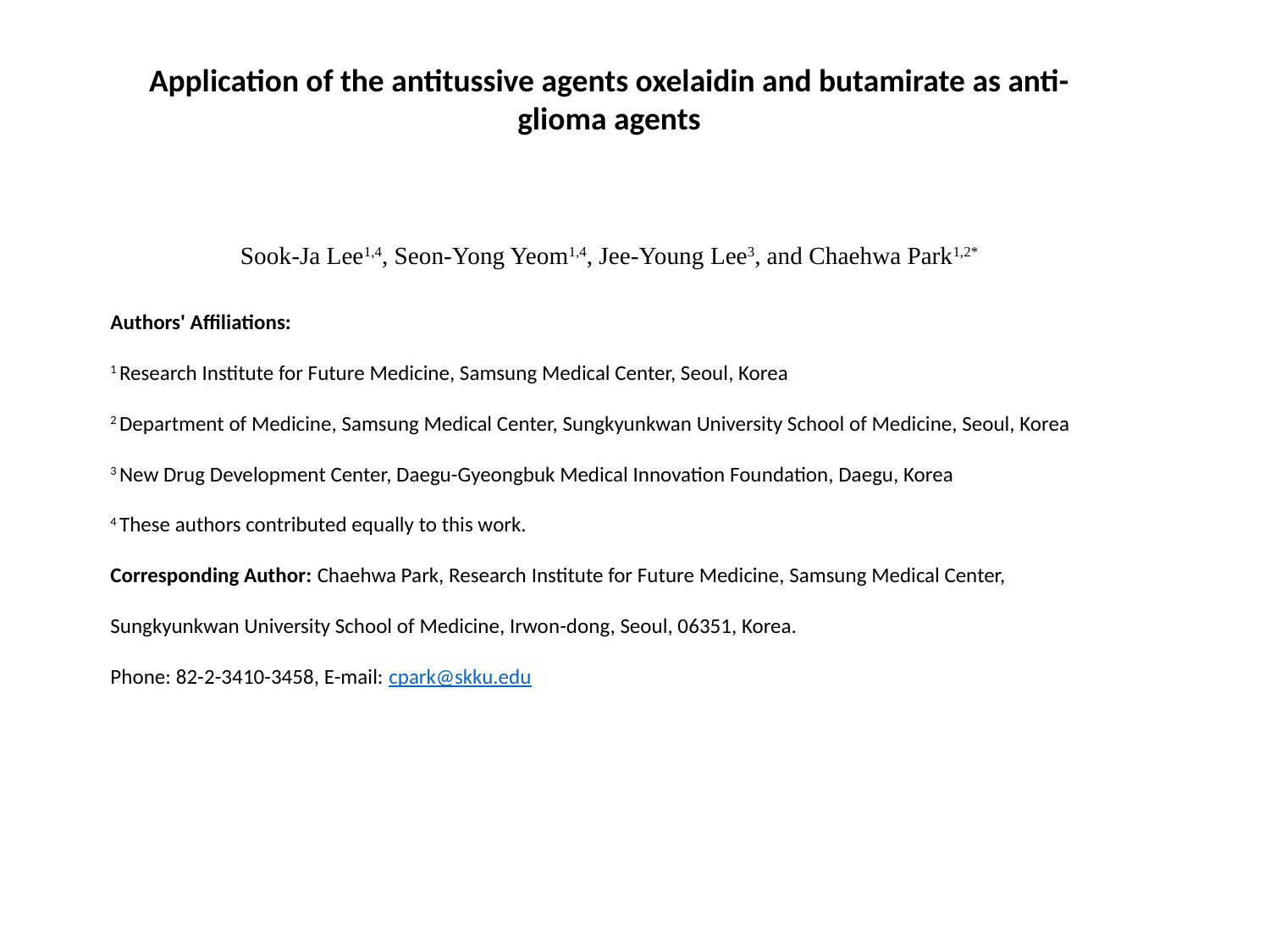

Application of the antitussive agents oxelaidin and butamirate as anti-glioma agents
Sook-Ja Lee1,4, Seon-Yong Yeom1,4, Jee-Young Lee3, and Chaehwa Park1,2*
Authors' Affiliations:
1 Research Institute for Future Medicine, Samsung Medical Center, Seoul, Korea
2 Department of Medicine, Samsung Medical Center, Sungkyunkwan University School of Medicine, Seoul, Korea
3 New Drug Development Center, Daegu-Gyeongbuk Medical Innovation Foundation, Daegu, Korea
4 These authors contributed equally to this work.
Corresponding Author: Chaehwa Park, Research Institute for Future Medicine, Samsung Medical Center, Sungkyunkwan University School of Medicine, Irwon-dong, Seoul, 06351, Korea.
Phone: 82-2-3410-3458, E-mail: cpark@skku.edu

## Slide 2
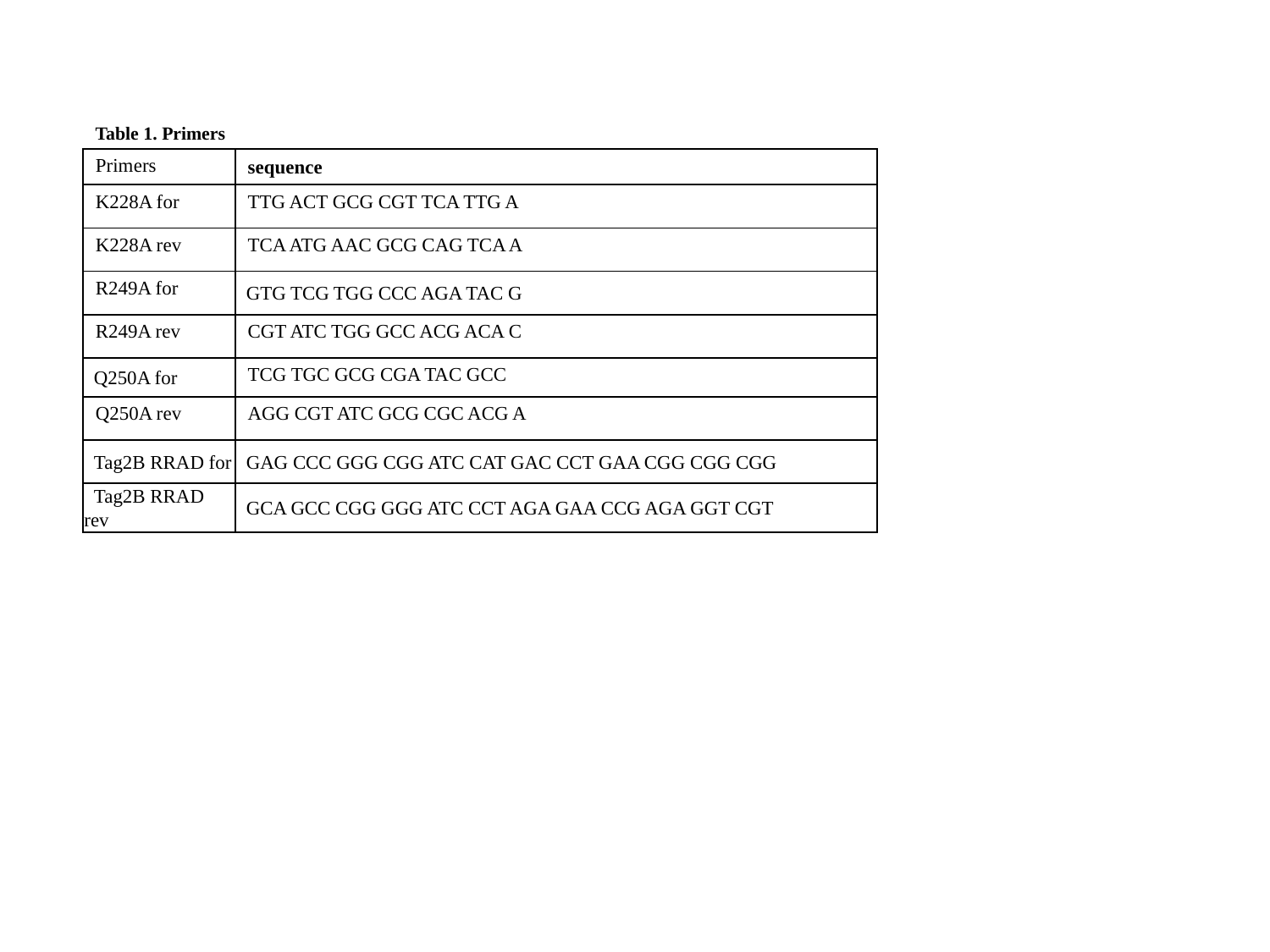

Table 1. Primers
| Primers | sequence |
| --- | --- |
| K228A for | TTG ACT GCG CGT TCA TTG A |
| K228A rev | TCA ATG AAC GCG CAG TCA A |
| R249A for | GTG TCG TGG CCC AGA TAC G |
| R249A rev | CGT ATC TGG GCC ACG ACA C |
| Q250A for | TCG TGC GCG CGA TAC GCC |
| Q250A rev | AGG CGT ATC GCG CGC ACG A |
| Tag2B RRAD for | GAG CCC GGG CGG ATC CAT GAC CCT GAA CGG CGG CGG |
| Tag2B RRAD rev | GCA GCC CGG GGG ATC CCT AGA GAA CCG AGA GGT CGT |

## Slide 3
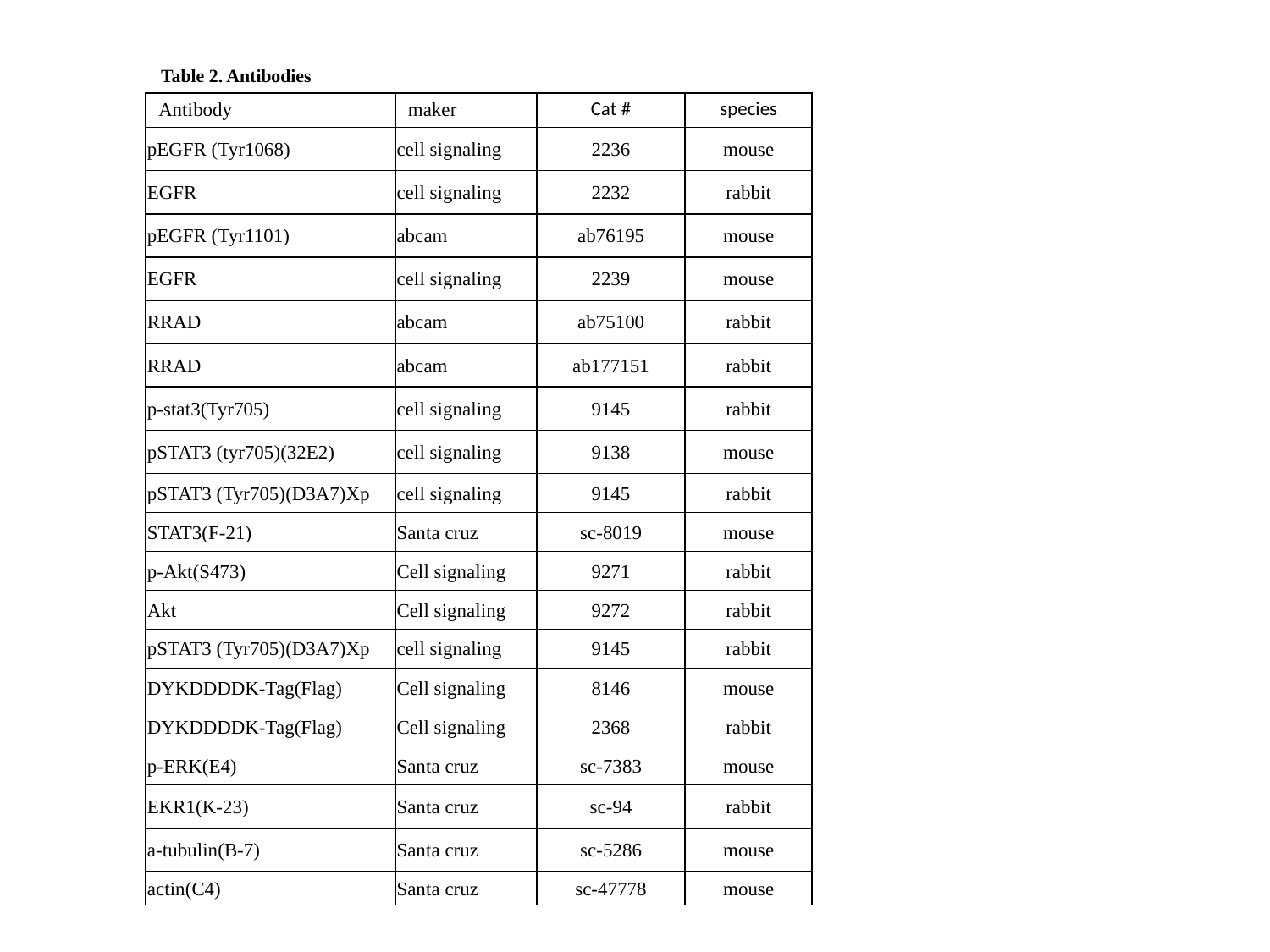

Table 2. Antibodies
| Antibody | maker | Cat # | species |
| --- | --- | --- | --- |
| pEGFR (Tyr1068) | cell signaling | 2236 | mouse |
| EGFR | cell signaling | 2232 | rabbit |
| pEGFR (Tyr1101) | abcam | ab76195 | mouse |
| EGFR | cell signaling | 2239 | mouse |
| RRAD | abcam | ab75100 | rabbit |
| RRAD | abcam | ab177151 | rabbit |
| p-stat3(Tyr705) | cell signaling | 9145 | rabbit |
| pSTAT3 (tyr705)(32E2) | cell signaling | 9138 | mouse |
| pSTAT3 (Tyr705)(D3A7)Xp | cell signaling | 9145 | rabbit |
| STAT3(F-21) | Santa cruz | sc-8019 | mouse |
| p-Akt(S473) | Cell signaling | 9271 | rabbit |
| Akt | Cell signaling | 9272 | rabbit |
| pSTAT3 (Tyr705)(D3A7)Xp | cell signaling | 9145 | rabbit |
| DYKDDDDK-Tag(Flag) | Cell signaling | 8146 | mouse |
| DYKDDDDK-Tag(Flag) | Cell signaling | 2368 | rabbit |
| p-ERK(E4) | Santa cruz | sc-7383 | mouse |
| EKR1(K-23) | Santa cruz | sc-94 | rabbit |
| a-tubulin(B-7) | Santa cruz | sc-5286 | mouse |
| actin(C4) | Santa cruz | sc-47778 | mouse |

## Slide 4
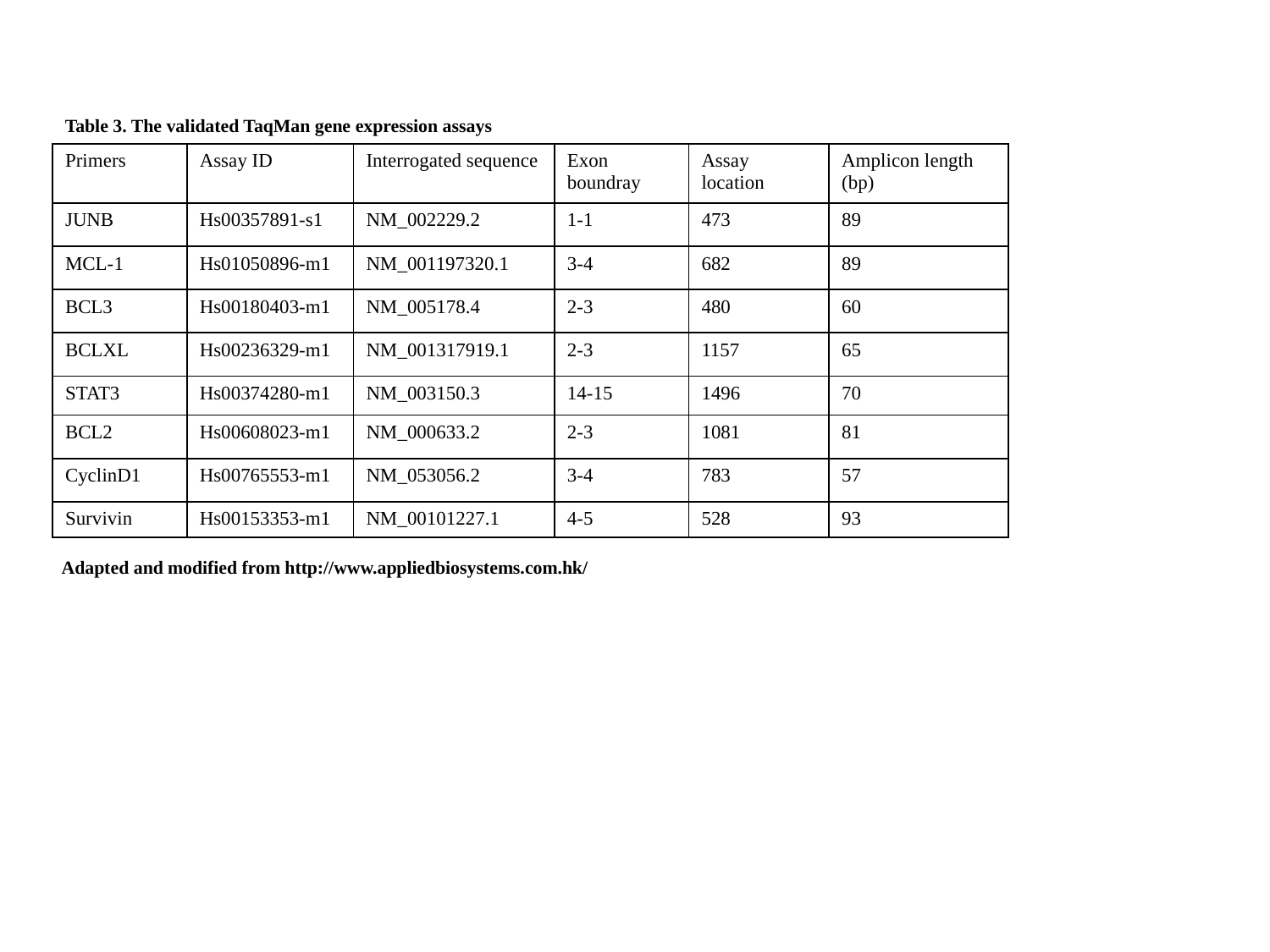

Table 3. The validated TaqMan gene expression assays
| Primers | Assay ID | Interrogated sequence | Exon boundray | Assay location | Amplicon length (bp) |
| --- | --- | --- | --- | --- | --- |
| JUNB | Hs00357891-s1 | NM\_002229.2 | 1-1 | 473 | 89 |
| MCL-1 | Hs01050896-m1 | NM\_001197320.1 | 3-4 | 682 | 89 |
| BCL3 | Hs00180403-m1 | NM\_005178.4 | 2-3 | 480 | 60 |
| BCLXL | Hs00236329-m1 | NM\_001317919.1 | 2-3 | 1157 | 65 |
| STAT3 | Hs00374280-m1 | NM\_003150.3 | 14-15 | 1496 | 70 |
| BCL2 | Hs00608023-m1 | NM\_000633.2 | 2-3 | 1081 | 81 |
| CyclinD1 | Hs00765553-m1 | NM\_053056.2 | 3-4 | 783 | 57 |
| Survivin | Hs00153353-m1 | NM\_00101227.1 | 4-5 | 528 | 93 |
Adapted and modified from http://www.appliedbiosystems.com.hk/
